# Prevalence of youth type 2 diabetes in global Indigenous populations: a systematic review

**DOI:** 10.1007/s00125-025-06556-7

**Published:** 2025-10-02

**Authors:** Emily R. Papadimos, Courtney Claussen, Dianna J. Magliano, Cheri Hotu, Alex Brown, Odette Pearson, Donald Warne, Louise Maple-Brown, Baiju R. Shah, Hiliary Monteith, Louise A. Baur, Andrew Cotterill, Anthony J. Hanley, Elizabeth L. M. Barr

**Affiliations:** 1 Menzies School of Health Research, Charles Darwin University, Darwin, NT Australia; 2 Department of Endocrinology and Diabetes, Queensland Children’s Hospital, Brisbane, QLD Australia; 3 Cheyenne River Lakota, School of Medicine and Health Sciences, University of North Dakota, Grand Forks, ND USA; 4 Johns Hopkins Bloomberg School of Public Health, Baltimore, MD USA; 5 Baker Heart and Diabetes Institute, Melbourne, VIC Australia; 6 School of Public Health and Preventive Medicine, Monash University, Melbourne, VIC Australia; 7 Ngāti Maniapoto, Ngāti Ruanui, Te Toka Tumai Auckland City Hospital, Te Whatu Ora, Aotearoa, New Zealand; 8 Yuin Nation, National Centre for Indigenous Genomics, Australian National University, Adelaide, SA Australia; 9 The Kids Research Institute Australia, Adelaide, SA Australia; 10 Eastern Kuku-Yalanji and Zenadth Kes (Torres Strait Islander), South Australian Health and Medical Research Institute, University of Adelaide, Adelaide, SA Australia; 11 Faculty of Health and Medical Sciences, University of Adelaide, Adelaide, SA Australia; 12 Oglala Lakota, Johns Hopkins Bloomberg School of Public Health, Baltimore, MD USA; 13 Endocrinology Department, Royal Darwin Hospital, Darwin, NT Australia; 14 Department of Medicine and Institute for Health Policy, Management and Evaluation, University of Toronto, Toronto, ON Canada; 15 Department of Nutritional Sciences, University of Toronto, Toronto, ON Canada; 16 Sydney Medical School, The University of Sydney, Sydney, NSW Australia; 17 Department of Medicine and the Dalla Lana School of Public Health, University of Toronto, Toronto, ON Canada; 18 Leadership Sinai Centre for Diabetes, Mt. Sinai Hospital, Toronto, ON Canada

**Keywords:** Adolescent, Children, Diabetes, Health equity, Indigenous health, Paediatrics, Prevalence, Systematic review, Type 2 diabetes, Youth

## Abstract

**Aims/hypothesis:**

We aimed to synthesise global prevalence estimates of type 2 diabetes among Indigenous youth aged under 25 years, and examine age- and gender-specific differences and secular trends.

**Methods:**

We searched MEDLINE, Embase, CINAHL and Cochrane, and bibliographies of included studies, from 1 January 1980 to 14 September 2024. We included cross-sectional observational studies that reported diabetes point prevalence estimates (per 1000) and prevalence trends in Indigenous youth aged under 25 years from all regions. Age- and gender-specific analysis and secular trends were reported. Study quality was assessed using a modified Newcastle–Ottawa Scale adapted for Indigenous health research.

**Results:**

From 2342 records and 27 additional references, 49 studies were retained for data extraction. Total type 2 diabetes prevalence, reported in 33 of 49 studies from 36 distinct populations across six countries and two self-governing states, varied widely (0–44 per 1000), with 75% (27/36) of the populations reporting a prevalence of over 1 per 1000. Age-specific data, available in 44 studies, showed increased prevalence with age: 0–4 per 1000 at age <10 years; 0–44 per 1000 at age 10–19 years; and 0–64 per 1000 at age 15–25 years. Of 22 studies with gender-specific data, 77% showed a female predominance. Secular trends, examined in 12 studies since 1981, showed a rising prevalence in young adults (aged 15–25 years) in eight of ten studies, and in youth aged under 15 years in six of nine studies. Heterogeneity in study design, diagnostic criteria, and incomplete age- and gender disaggregation precluded meta-analysis.

**Conclusions/interpretation:**

Youth type 2 diabetes prevalence in Indigenous populations is very high, particularly in young adulthood, and among the female sex. Prevalence has increased over time. Future research should stratify data by age and pubertal status, and identify both protective and risk factors to inform targeted prevention strategies. Indigenous-led, community-specific approaches that actively engage youth are critical in the development and implementation of diabetes surveillance, prevention and management programmes.

**Trial registration:**

PROSPERO registration no. CRD42021278418.

**Graphical Abstract:**

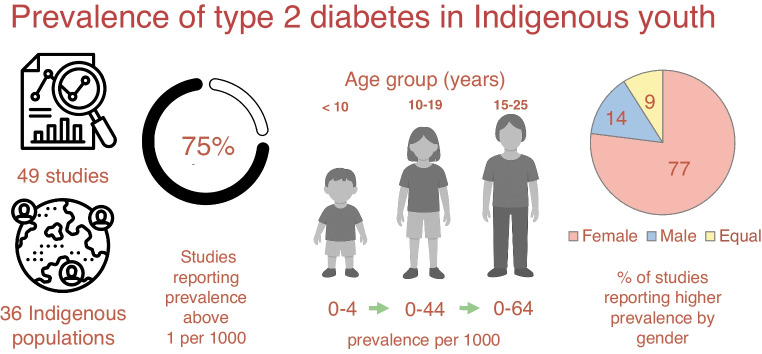

**Supplementary Information:**

The online version of this article (10.1007/s00125-025-06556-7) contains peer-reviewed but unedited supplementary material.


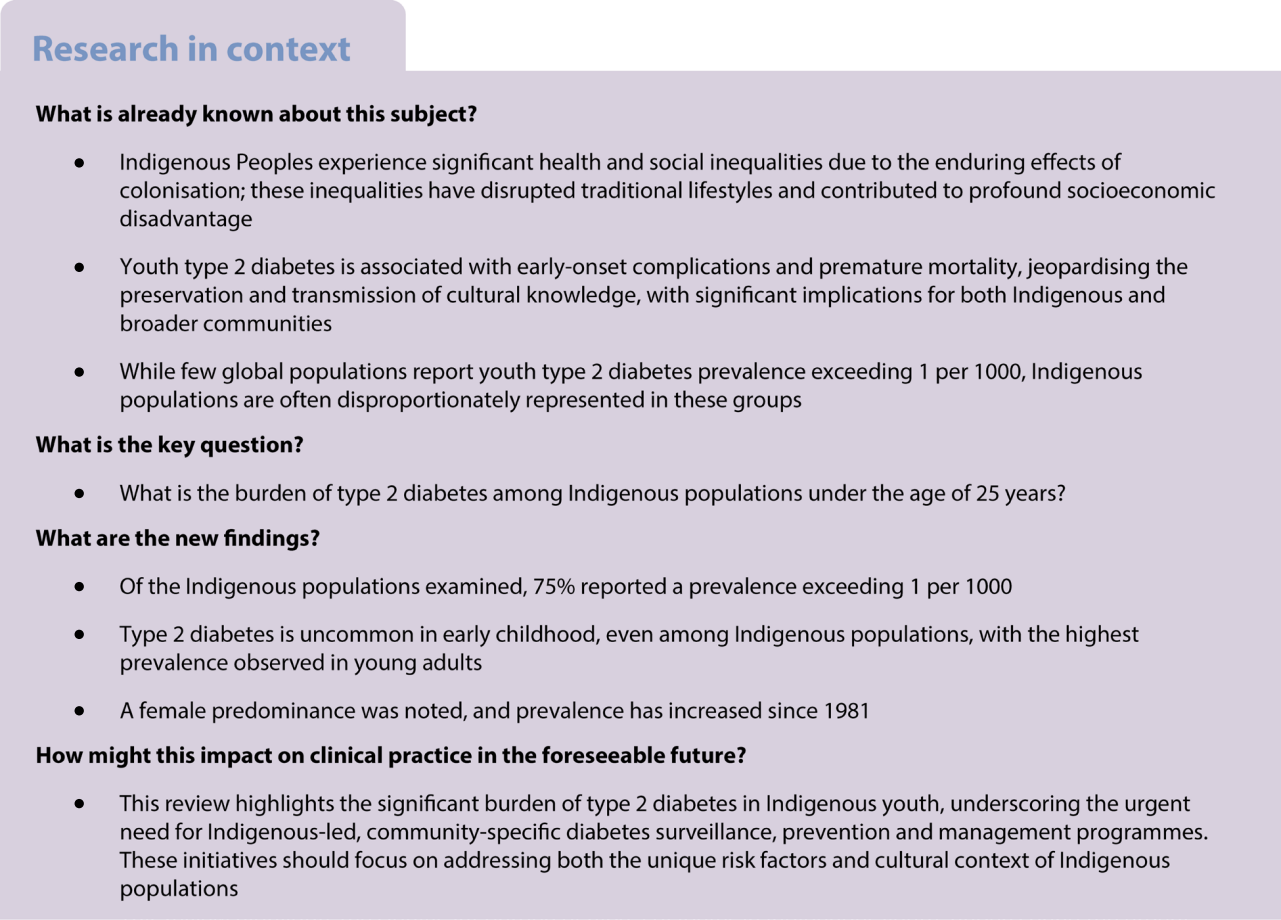


## Introduction

Type 2 diabetes in young people (defined here as <25 years of age or ‘youth type 2 diabetes’) is associated with adverse trajectories of diabetes-related complications and premature mortality [1, 2]. Emerging evidence suggests that complication rates are higher than in those diagnosed in adulthood and in those with type 1 diabetes, even after adjustment for age [1, 3].

Type 2 diabetes disproportionately affects Indigenous youth, who often experience socioeconomic and psychological adversity [3]. The term ‘Indigenous’ reflects a colonial perspective. In this article, we use the United Nations Permanent Issues Forum on Indigenous Issues framework when referring to ‘Indigenous People’ [4] wherein self-determination underscores the right of Indigenous People to uphold their unique and diverse cultures. The ongoing and transgenerational impacts of colonisation vary between Indigenous populations but collective experiences of forced displacement from traditional territories, disruption to cultural practices and traditional food systems, enduring trauma and structural racism contribute to significant health disparities between Indigenous and non-Indigenous Peoples [5]. In addition to Indigenous-specific social determinants of health, the Developmental Origins of Health And Disease (DOHAD) theory links genetics, in utero exposures, nutrition, physical activity, weight gain and body fat distribution to increased risk of cardiometabolic disease, including type 2 diabetes [3]. Premature diabetes-related mortality threatens the transmission of cultural knowledge to future generations, with significant implications for both Indigenous and broader communities. Understanding the burden and trends of type 2 diabetes in Indigenous youth is crucial for prioritising resources for effective diabetes surveillance, management and prevention. Throughout the manuscript, we use the term ‘type 2 diabetes’, acknowledging that diabetes subtypes are not always clearly defined in the literature. However, this terminology reflects the relative infrequency (2–7%) of type 1 diabetes among Indigenous youth [6, 7].

Although evidence points to a type 2 diabetes crisis among Indigenous communities, global prevalence among Indigenous youth has not been comprehensively analysed. A 2020 narrative review summarised youth-onset type 2 diabetes prevalence data from 12 countries and reported the highest prevalence in China (5.2 per 1000) and the lowest in Denmark (0.006 per 1000) [8]. The 10th edition of the IDF Diabetes Atlas reported a youth-onset type 2 diabetes prevalence below 1 per 1000 in most non-Indigenous populations [6, 9]. In contrast, specific Indigenous populations and regions, including Akimel O’odham and Tohono O’odham (formerly referred to as Pima Indian and Papago, respectively) youth aged 15–24 years in the Gila River Indian Community (64 per 1000) [10] and First Nations youth aged 15–24 years in Central Australia (31 per 1000), showed much higher prevalence [11].

This systematic review aims to describe the total, age- and gender-specific prevalence and secular trends in type 2 diabetes prevalence among global Indigenous populations aged under 25 years.

## Methods

### Search strategy and selection criteria

This review followed PRISMA 2020 guidelines [12] and was registered with PROSPERO (https://www.crd.york.ac.uk/PROSPERO/view/CRD42021278418) on 13 October 2021. Three protocol deviations were made during data extraction to enhance data consistency, cultural relevance and rigour. First, the upper age cut-off was refined from <30 years to <25 years to align with the age groupings used in most included studies and facilitate data synthesis. Second, we selected the Newcastle–Ottawa Scale (NOS) for risk of bias assessment, as it offered greater flexibility to incorporate culturally relevant criteria, including Indigenous population representation. Third, we added specific exclusion criteria to omit studies that combined Indigenous and non-Indigenous data when disaggregation was not possible. The manuscript incorporates Indigenous-specific culturally appropriate publishing recommendations [13]. MEDLINE, Embase, CINAHL and Cochrane database were searched to identify cross-sectional studies published from 1 January 1980 to 14 September 2024 to collect articles that were published after the diabetes diagnostic criteria were changed in 1979/1980. The search strategy was developed by the Diabetes in Indigenous Populations Special Interest Group of the International Diabetes Federation Atlas Working Group, a diverse authorship group aimed to balance sensitivity with specificity. The ‘United Nations Permanent Forum on Indigenous Issues’ criteria [4] and author expertise guided the identification of Indigenous populations. Full search strategies and members of the Diabetes in Indigenous Populations Special Interest Group of the International Diabetes Federation Atlas Working Group are provided in the electronic supplementary material (ESM) Methods. To improve the search strategy sensitivity, we reviewed the reference lists of included studies and relevant narrative reviews to identify additional eligible publications. See ESM Fig. 1 for the PRISMA selection flow chart and ESM Table 1 for details of included studies. After the removal of duplicates, titles, abstracts and full-text papers were screened by two independent reviewers (ERP, CC, DJM, CH, HM, BRS, AJH, ELMB) using a web-based collaborative software platform, Covidence (Melbourne, Australia) [14]. Disagreements were resolved by consensus of a third reviewer (ERP, CC, DJM). Diabetes prevalence from cross-sectional studies was extracted for the total population, region and age- and gender-specific groups; the most recent data was used when multiple studies reported on the same cohort or population.

#### Inclusion criteria

Studies that included data for Indigenous children or young people aged ≤25 years from any geographical region, reporting the prevalence of type 2 diabetes or unspecified diabetes (including undiagnosed diabetes), were eligible for inclusion. Eligible study designs included cross-sectional studies using population-based data or health databases (e.g. administrative datasets, medical records, registries). The <25 years age criterion used in this review overlapped with that of a concurrent adult-focused systematic review conducted by our group [15].

#### Exclusion criteria

Studies were excluded based on the following criteria:

- data could not specifically be extracted for individuals aged ≤25 years
- Indigenous and non-Indigenous data were combined without disaggregation
- the study explicitly reported on type 1 diabetes or MODY
- the abstract or full text was unavailable
- the study lacked clear methodological description
- prevalence was reported in specific cohorts (e.g. occupations, specific diseases)
- the study was not in English
- the study involved non-humans

### Data extraction and analysis

Data extraction was performed by two reviewers (ERP, ELMB) and recorded in a pre-specified data extraction template using Microsoft Excel version 16.78.3 (ESM Table 1). Inconsistencies were resolved through consensus among authors. Missing data were requested from authors where possible. Prevalence was reported per 1000 Indigenous youth, and total, age-specific and gender-specific data were arranged in descending order. Prevalence reported as a percentage was converted to prevalence per 1000. Age-specific prevalence data were extracted from the age range provided in each study. Age groups were categorised into pre-defined ranges approximating likely pubertal stages: child (age <10 years); adolescent (10–19 years); and young adult (15–25 years). When multiple studies reported on the same broad population group, the most recent data were included in the cross-sectional prevalence analyses to avoid duplication. To analyse prevalence trends, only studies that explicitly reported data from the same Indigenous population using consistent methodology across at least two time points were included. Where possible, guidance was provided by authors with expertise and knowledge of specific Indigenous populations and Tribal Nations across regions. Trends data were stratified into three age groups (<15 years, 15–24 years and a heterogenous group encompassing ages 7–19 years) and examined in intervals of 5 years. Crude and standardised prevalence estimates were extracted and, for standardised estimates, the populations used in the analysis were recorded. Where results were not offered in text, raw data were extracted from graphs using Bormann I DigitizeIt Software, version 2.5 (https://www.digitizeit.de, accessed 29 September 2024).

#### Quality assessment

Study quality was assessed by two independent reviewers (ERP, CC, DJM, AJH, ELMB) using a modified Newcastle–Ottawa Quality Assessment Scale [16], adapted to reflect key features of Indigenous populations (ESM Fig. 2). The maximum score was 10 (low, 0–3; medium, 4–7; high, ≥8). Studies were not excluded based on the quality assessment; however, the scores are provided.

#### Indigenous governance

Indigenous governance in this study was provided by the Indigenous expertise of the authors (CC, CH, AB, OP, DW) who had lived experience of the impact that type 2 diabetes has on their communities and families. We were committed to providing collective Indigenous governance based on high-level principles across all areas of the review method, recognising that we do not hold knowledge on the diversity of the populations who gifted their data to respective studies [17, 18]. We acknowledge the important contributions by young people from each Indigenous population included in this systematic review. Translation of cultural knowledge to future generations is vital.

## Results

### Characteristics of the included studies

The database search identified 2342 studies after removal of duplicates, published between 1980 and 2024, with an additional 27 studies added from the bibliography review. After title and abstract screening, 184 studies were reviewed in full to assess eligibility and 49 studies were retained for data extraction (ESM Fig. 1). Data from the included studies were collected between 1978 and 2019 from six countries and two self-governing states (USA, Canada, Australia, Aotearoa New Zealand, Nauru, Argentina, the Cook Islands and Niue) and at least 45 Indigenous populations. The exact number of Indigenous populations could not be determined as several studies included Indigenous populations from multiple regions, where details of specific populations were incomplete. A summary of the study characteristics for which data were extracted is available in ESM Table 1. Canada had the highest number of studies (*n*=17, 35%), followed by the USA (*n*=15, 31%). Data were derived equally from population-based screening (*n*=26, 53%) and health databases (*n*=23, 47%). Studies used a range of methods to diagnose and classify diabetes, reflecting changing international and region-specific guidelines over time (ESM Table 1). Diabetes classification was specifically defined as type 2 diabetes in 25 of 49 studies (51%), and the remaining studies did not specify diabetes subtypes. Data sources included health databases (ICD, registries or electronic medical records) or population-based screening. Diagnostic methods included HbA_1c_, fasting glucose, random glucose, 2 h 75 g OGTT or self-report. Age group definitions varied between studies. Only two studies reported on pubertal stage: one based on parental report [19]; and one using sex-steroid levels [20]. Crude prevalence estimates were reported in all but three studies (that reported age- or gender-standardised estimates), which are detailed in ESM Table 1.

### Type 2 diabetes prevalence by total population aged <25 years

**Fig. 1:**
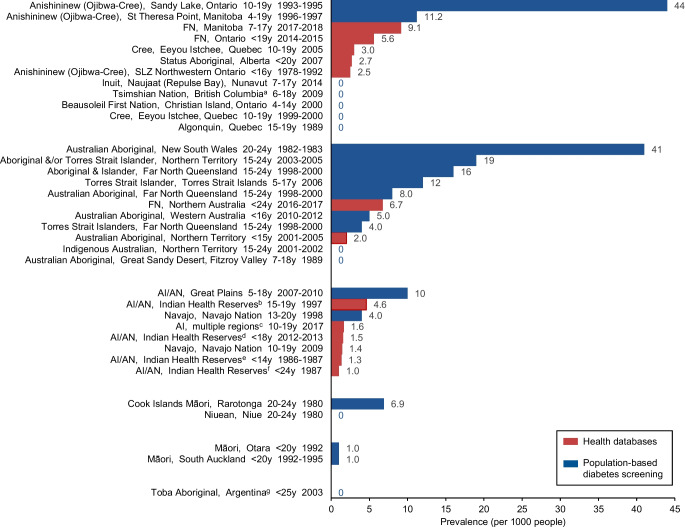
Total population prevalence of type 2 diabetes among Indigenous youth by region and according to method of diabetes diagnosis, 1978–2018 (prevalence per 1000 people). Health databases include ICD coding, registries or electronic medical records. Population-based diabetes screening methods included 2 h 75 g OGTT, HbA_1c_, fasting plasma glucose, random plasma glucose or self-report. ^a^Gitga’at (Hartley Bay), Gitkxaahla (Kitkatla), Lax Kw’alaams (Port Simpson). ^b^Arizona, Colorado, Nevada, New Mexico & Utah. ^c^Four sites in Ohio; Colorado; five counties around Seattle, Washington; South Carolina; two sites in Hawaii and California; American Indian reservation-based populations in Arizona and New Mexico. ^d^Six Indian Health Service regions (Alaska, East, Northern Plains, Pacific Coast, Southern Plains, Southwest). ^e^432 Indian Health Service facilities across the USA. ^f^Washington, Oregon and Idaho. ^g^Toba, Mapic, Chelliyi & Fidelidad. AI, American Indian; AN, Alaskan Natives; FN, First Nations; SLZ, Sioux Lookout Zone; y, years

Type 2 diabetes prevalence across populations, regions and ages was reported from six countries and two self-governing states and is presented in Fig. 1 [6, 7, 11, 19–48]. Of the 49 studies, 33 reported total prevalence among those aged <25 years across 36 distinct Indigenous populations (one included three distinct Australian Indigenous populations and one included two distinct Indigenous populations in the Western Pacific region). Of the 36 distinct populations, 27 (75%) reported diabetes prevalence above 1 per 1000. These included eight studies involving distinct American Indian and Alaskan Native populations in the USA [6, 19, 39–44], seven studies including Aboriginal and/or Torres Strait Islander populations in Australia (one including three populations) [11, 31–36, 38], seven studies including Canadian First Nation populations [7, 21–26], and two studies involving Māori youth in Aotearoa New Zealand [46, 47] and Cook Island Māori youth in the Cook Islands [45]. Prevalence per 1000 ranged from 0 to 44 in population-based screening studies and from 0 to 9.1 in database studies (Fig. 1). Although both sources reported a similar proportion of studies with prevalence above 1 per 1000, population-based studies were more likely to report prevalence exceeding 10 per 1000. Nine population-based screening studies reported no diabetes (0% prevalence); these included studies in a Toba Aboriginal population in Argentina [48], two distinct Australian Aboriginal populations [37, 38], Niuean People [45], four Canadian First Nations populations (Cree [29], Algonquin [30], Tsimshian Nation [20] and Beausoleil First Nation Peoples [28]) and an Inuit population [27]. The highest prevalence was reported in population-based screening among Anishininew (Ojibwa-Cree) youth aged 10–19 years in Sandy Lake, Ontario [21]. Prevalence (per 1000) ranged from 0 to 44 in studies that specifically defined type 2 diabetes and from 0 to 19 in studies that did not specify diabetes subtype. Both groups reported similar proportions of studies with prevalence above 1 per 1000 (76% and 74%, respectively) and above 10 per 1000 (18% and 21%, respectively) (ESM Fig. 3).

### Age-specific type 2 diabetes prevalence

**Fig. 2:**
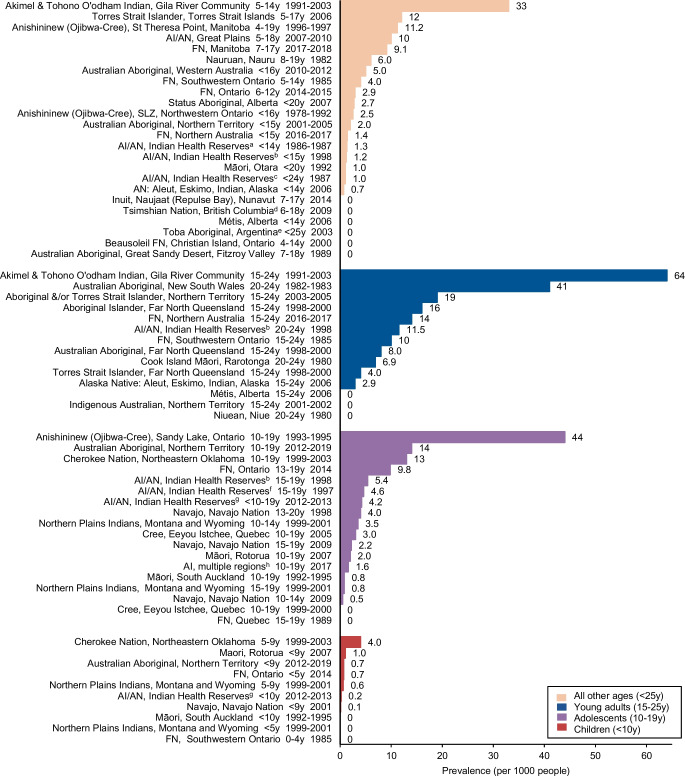
Prevalence of youth-onset type 2 diabetes among global Indigenous populations according to age range (prevalence per 1000 people). ^a^432 Indian Health Service facilities across the USA. ^b^Alaska, Great Lakes, Northern Plains, Pacific, Southeast, Southern Plains, Southwest. ^c^Washington, Oregon and Idaho. ^d^Gitga’at (Hartley Bay), Gitkxaahla (Kitkatla), Lax Kw’alaams (Port Simpson). ^e^Toba, Mapic, Chelliyi and Fidelidad. ^f^Arizona, Colorado, Nevada, New Mexico & Utah. ^g^Six Indian Health Service regions (Alaska, East, Northern Plains, Pacific Coast, Southern Plains, Southwest). ^h^Four sites in Ohio; Colorado; five counties around Seattle, Washington; South Carolina; two sites in Hawaii and California; American Indian reservation-based populations in Arizona and New Mexico. AI, American Indian; AN, Alaskan Natives; FN, First Nations; SLZ, Sioux Lookout Zone; y, years

Of the 49 studies, 44 (Fig. 2) [6, 7, 10, 11, 19–58] from six countries and two self-governing states reported age-specific prevalence. Type 2 diabetes prevalence was highest in Indigenous adolescents and young adults. In young adults (aged 15–25 years), type 2 diabetes prevalence was 0–64 per 1000, with the highest prevalence of type 2 diabetes in Akimel O’odham and Tohono O’odham Peoples from the Gila River Indian Community [10]. Three populations reported no diabetes: Niuean People [45]; Métis Indigenous people of Canada [52]; and Indigenous Australians in a remote region of the Northern Territory [37]. In adolescents (aged 10–19 years), type 2 diabetes prevalence was 0–44 per 1000, with the highest prevalence in Anishininew (Ojibwa-Cree) youth from Sandy Lake, Ontario [21] and no diabetes reported in two populations (First Nations in Quebec [30] and Cree Peoples from Eeyou Istchee [29]). In those aged <10 years, type 2 diabetes prevalence was 0–4 per 1000, with the highest prevalence reported in Cherokee Nation children [55] and no diabetes reported in three populations (Māori children in South Auckland, Northern Plains Indians from Montana and Wyoming [56] and First Nations Peoples from Southwestern Ontario [53]).

### Gender-specific type 2 diabetes prevalence

**Fig. 3:**
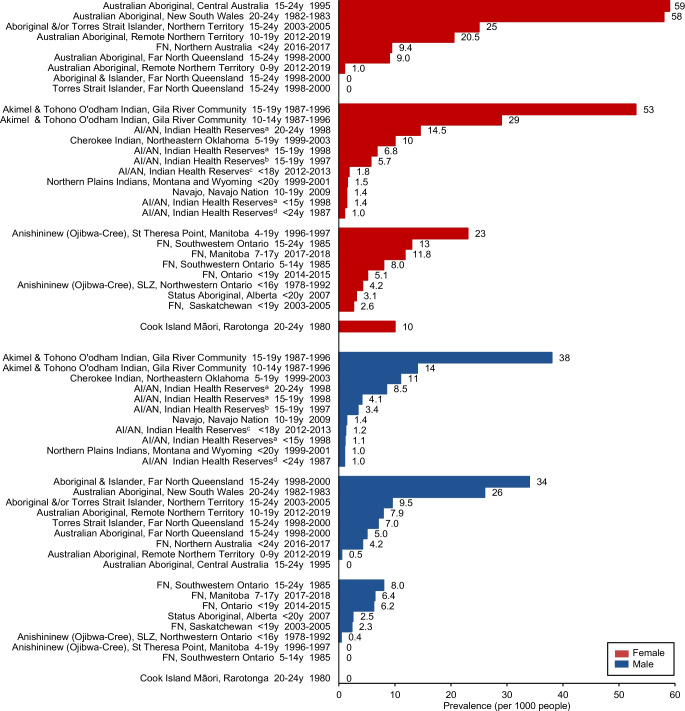
Prevalence of youth type 2 diabetes from global Indigenous populations according to region and gender (prevalence per 1000). ^a^Alaska, Great Lakes, Northern Plains, Pacific, Southeast, Southern Plains, Southwest. ^b^Arizona, Colorado, Nevada, New Mexico & Utah. ^c^Six Indian Health Service regions (Alaska, East, Northern Plains, Pacific Coast, Southern Plains, Southwest). ^d^Washington, Oregon and Idaho. AI, American Indian; AN, Alaskan Natives; FN, First Nations; SLZ, Sioux Lookout Zone; y, years

Gender-specific differences were reported in 22 of the 49 studies (Fig. 3) [7, 22, 23, 25, 26, 31–33, 39, 41, 42, 44, 45, 50, 53–56, 59–61] from three countries and a self-governing state (USA, Canada, Australia and the Cook Islands). Of these, 17 (77%) reported female predominance, three studies (14%) reported male predominance and two studies (9%) reported similar prevalence in both genders. The greatest gender difference was observed in a screening study of primary healthcare records involving 97 Australian Aboriginal youth aged 15–24 years in Central Australia [59].

### Type 2 diabetes prevalence trends

**Fig. 4:**
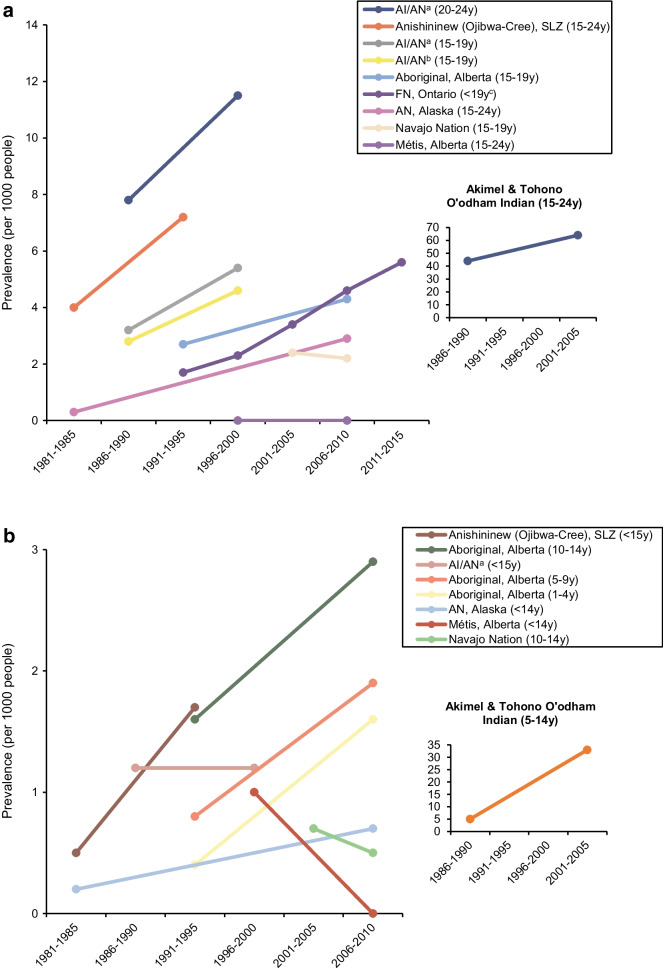
Type 2 diabetes prevalence trends in Indigenous youth, 1981–2015 (prevalence per 1000). (**a**) Age 15–24 years, 1981–2015. (**b**) Age under 15 years, 1981–2010. ^a^Alaska, Great Lakes, Northern Plains, Pacific, Southeast, Southern Plains, Southwest. ^b^Arizona, Colorado, Nevada, New Mexico and Utah. ^c^>78% of participants age 13–19 years. AI, American Indian; AN, Alaskan Native; FN, First Nations; SLZ, Sioux Lookout Zone; y, years

Few studies (12/49; 25%) reported prevalence over time, with data limited to Canada and the USA. Data are presented for those aged 15–24 years, <15 years (Fig. 4) [23, 25, 39, 42, 50–52, 62, 63], and all ages combined (ESM Fig. 4) [6, 7]. Data from two studies among those aged 7–19 years are presented separately, as participants crossed both age groups (ESM Fig. 5). Of the data available, an increasing trend over time, particularly in young adults (aged 15–24 years) was observed. The increase in prevalence for Akimel O’odham and Tohono O’odham Indian youth [10] was greater than that observed for the other populations reporting trend data, particularly in those aged <15 years, where diabetes prevalence increased more than eightfold over two decades. Data from Navajo youth participants in the SEARCH study showed stable prevalence between 2001 and 2009 [42]. Type 2 diabetes prevalence in Métis children aged <15 years declined between 1998 and 2006 [52].

#### Quality assessment

Of the studies included, 33% (*n*=16/49), 67% (*n*=33/49) and 0% were assessed as being high, medium and low quality, respectively. Further details of the quality assessment are presented in ESM Table 2.

## Discussion

This comprehensive systematic review demonstrates an extremely high prevalence of type 2 diabetes among Indigenous youth, with considerable regional and population variation. Of the Indigenous populations reporting total population prevalence, 75% reported a prevalence above 1 per 1000, compared with only 17% among non-Indigenous youth as reported in the IDF Diabetes Atlas [9]. Since the first case reports in 1981 [64], youth type 2 diabetes threatens to pose significant health challenges, and disproportionally affects Indigenous populations. Our review shows that prevalence was highest among young adults (most likely to be post-pubertal) and lowest among children (most likely to be pre-pubertal), and higher in female compared with male populations, consistent with findings reported in Indigenous adult populations [15]. Most populations showed an increase in prevalence over time since 1981.

Previous reviews of type 2 diabetes prevalence in youth have focused on region- [65] or country-specific data [8, 9], with few including Indigenous populations despite the considerable health disparities [5]. Although Indigenous Peoples are estimated to inhabit more than 90 countries [4], and collectively represent over 370 million people, the 49 studies included in this systematic review involve populations from only six countries and two self-governing states. Despite over 80% of the world’s Indigenous Peoples residing in Asia, Latin America and Africa [4], no studies from these regions met our inclusion criteria. Nine populations in the included studies reported no evidence of diabetes. Regional differences in diabetes prevalence may reflect variation in healthcare access, screening practices and uptake of established registries in these regions [66]. Methodological differences in study design likely contribute to heterogeneity in prevalence estimates. Health database studies may underestimate true prevalence due to missed undiagnosed cases and barriers to healthcare access, whereas population-based studies may yield higher estimates by actively identifying undiagnosed cases and reducing access-related bias. Alternatively, populations and regions with a low prevalence of youth type 2 diabetes may differ in socioecological factors, including preservation of traditional cultural practices, access to fresh and nutrient-rich food and safe access to physical activity, which may combine to mitigate rapid, excess weight gain and protect against the development of type 2 diabetes [66]. Recent timing of colonisation [37] and protective genetic or epigenetic factors may also explain the lower type 2 diabetes prevalence but empirical data to explore this contention does not yet exist. In studies that did not explicitly define the diabetes subtype, prevalence estimates were similar to those reporting type 2 diabetes specifically. This supports our decision to combine these data while acknowledging the potential for modest overestimation of type 2 diabetes prevalence due to possible inclusion of other subtypes.

Our data show that type 2 diabetes is uncommon in children aged <10 years, and prevalence increases with age, consistent with non-Indigenous populations [8]. Physiologically driven insulin resistance during puberty [67] may contribute to the increased prevalence of diabetes in this age group. However, the physiological differences in puberty between youth with and without diabetes need further examination. Epidemiological data show that maternal diabetes and obesity are associated with an increased risk of youth-onset diabetes in offspring [68–70], and accumulating evidence indicates that exposure to maternal obesity and diabetes may trigger epigenetic changes that enhance intergenerational diabetes risk. Among Indigenous populations, Tohono O’odham youth exposed to maternal diabetes in utero were nine times more likely to develop diabetes by age 24 years compared with non-exposed peers [69]. In a cohort of Canadian First Nations children born to mothers who had previously had youth type 2 diabetes, 43% of the children developed diabetes by age 19 years [71]. Compared with non-Indigenous peers born to mothers without diabetes, Canadian First Nations offspring exposed in utero to maternal type 2 diabetes were 50 times more likely to develop type 2 diabetes [68]. This is of particular significance given the mounting evidence that the burden and severity of diabetes complications are greater for those diagnosed at younger age [2], especially for Indigenous youth [72]. Among Canadian First Nations Peoples, the incidence of end-stage kidney disease was 2.8 times higher and the mortality rate was two times higher than non-Indigenous people with youth-onset type 2 diabetes, despite similar age at diagnosis and duration of disease [73]. These data suggest there may be differences in disease progression between populations. Resources to implement community-tailored diabetes prevention and management programmes for communities experiencing a high prevalence of youth type 2 diabetes is essential to reduce the burden of diabetes-related complications.

Most of our included studies reported that diabetes prevalence was higher in young female vs male participants, consistent with reports in non-Indigenous youth [11, 74]. This predominance is also observed in Indigenous adults [15], in contrast to global total adult population estimates suggesting predominance in men during midlife and equivalent prevalence for men and women in later life [9, 74]. The reasons for these gender-specific differences are unclear but may be related to the earlier timing of peak insulin resistance during puberty, differences in weight gain and distribution, or varying physical activity levels during adolescence, possibly leading to higher obesity prevalence in female vs male adolescents [1, 74]. Data on pubertal stage were insufficient to formally assess its associations with diabetes prevalence. Earlier diagnosis due to universal screening recommendations in pregnancy and post-partum may also contribute to the observed gender difference, although evidence is lacking.

Of the secular trends data available, the prevalence increased in most of the included Indigenous populations. Some populations showed a more rapid rise, possibly due to methodological differences in diabetes diagnosis. This may account for some variability between reported prevalence rates. Although not statistically analysed, there appeared to be a higher prevalence in studies that used OGTT, a more sensitive screening method than fasting glucose or HbA_1c_ [75]. Thus, our findings may underreport the true burden. Variation in prevalence may also reflect differences in community and healthcare provider awareness, accessibility of screening programmes and healthcare, and youth health-seeking behaviours. Differences in socioecological and cultural protective factors between populations may partly explain variations over time or between populations. The rise in type 2 diabetes prevalence over the past decades has occurred in parallel with the rise in obesity among children and adolescents [3]. Although we did not specifically assess obesity rates in this study, future research into the correlation between obesity and youth type 2 diabetes is needed.

This is the first comprehensive review of youth type 2 diabetes prevalence among Indigenous populations. Several limitations should be considered. First, self-reported ethnicity is subject to misclassification [5] and health data for Indigenous Peoples are often incomplete due to ongoing inequities brought on by colonisation [5]. Although we aimed to be inclusive and respectful in our approach, some populations and studies may have been inadvertently omitted. The lack of studies from Asia, Latin America or Africa may reflect the exclusion of non-English publications, limited data disaggregation by Indigenous status or under-resourced health systems in some regions. Second, inclusion of studies that did not specify diabetes subtype may have led to some overestimation. However, prevalence estimates were similar between studies that did and did not define diabetes subtype. While type 1 diabetes is the most common form globally among youth [9], it represents only 2–7% of cases among Indigenous youth [6, 7], suggesting minimal impact on overall estimates. Third, heterogeneity in diagnostic criteria across studies precluded quantitative data synthesis and likely contributed to variation in reported prevalence. Fourth, some studies had small sample size, possibly reducing precision and affecting age- or gender-specific estimates. Fifth, type 2 diabetes may remain asymptomatic for years [3, 76]. The misalignment and failure of healthcare systems and services to meet the specific needs of Indigenous youth contributes to missed opportunities for early diabetes diagnosis [76–78]. Indigenous-led primary health services address many of the sociocultural determinants of health, ensuring culturally safe and accessible healthcare for Indigenous Peoples. Sixth, since existing risk of bias tools did not adequately assess cultural and methodological factors relevant to Indigenous populations, Indigenous researchers within the author group developed a version of the NOS to address these domains. We recognise that other quality assessment tools may have produced different results. However, as our assessment was primarily descriptive and not intended to exclude any studies, this is unlikely to have impacted our findings. Although not formally validated, the tool demonstrated high inter-rater reliability (92%) and reflected key principles of Indigenous-led evaluation. Finally, despite a comprehensive search strategy and hand-searching of references, some studies may have been missed due to database indexing or limited sensisitivity of the search strategy. We also acknowledge the under-representation of studies from regions such as Africa and Asia, and recommend that future research prioritise the inclusion of populations from these areas.

### Conclusion

This comprehensive systematic review of studies reporting type 2 diabetes prevalence among Indigenous Peoples aged <25 years globally identified a very high prevalence of type 2 diabetes among Indigenous youth, particularly female youth, with prevalence increasing with age and rising in most populations since 1981. Urgent action is needed to improve data equity through the inclusion of Indigenous populations in health surveillance, routine disaggregation by Indigenous status, and culturally safe research partnerships led by Indigenous communities. Standardised age group classifications, age- and gender-specific reporting, and assessment of comorbid obesity are essential to define healthcare needs and identify regions that would benefit from enhanced early detection and management. Understanding disease drivers such as disruption to culture, impacts of colonisation, in utero exposures, genetic and epigenetic influences and socioeconomic and sociocultural determinants is critical to informing effective prevention strategies. Epidemiological studies that include Indigenous data must uphold governance frameworks that prioritise Indigenous data sovereignty principles and practices, ensuring continuous improvements in research conduct. Indigenous-led, culturally respectful and community-tailored approaches that actively engage youth are essential for developing and implementing effective diabetes screening, surveillance, prevention and management programmes.

## Supplementary Information

Below is the link to the electronic supplementary material.

Supplementary file1 (PDF 766 KB)

## Data Availability

All data generated or analysed during this study are included in the ESM.

## Abbreviation

NOS: Newcastle–Ottawa Scale
