# Supplementary material for: Prevalence of youth type 2 diabetes in global Indigenous populations: a systematic review
Source: Diabetologia. 2025 Oct 2;69(3):568–81. doi: 10.1007/s00125-025-06556-7 (PMC12881184; doi:10.1007/s00125-025-06556-7)
Supplement: Supplementary file 1 — Supplementary file1 (PDF 766 KB) [file 125_2025_6556_MOESM1_ESM.pdf]

Electronic supplementary material to:

Prevalence of youth type 2 diabetes in global Indigenous populations: a systematic review

Table of Contents: Electronic Supplementary Material (ESM)

ESM METHODS 2
Search strategy – MEDLINE and Embase 2
Search strategy – CINAHL 5
Diabetes in Indigenous Populations Special Interest Group of the International Diabetes Federation Diabetes Atlas Working Group Members 6
ESM Fig 1. PRISMA study selection flowchart 7
ESM Fig 2. Modified Newcastle-Ottawa Quality assessment scale 8
ESM TABLES 9
ESM Table 1. Data extraction 9
ESM Table 2. Modified Newcastle-Ottawa Quality assessment results 15
ESM FIGURES 18
ESM Fig 3. Diabetes prevalence according to diabetes subtype 18
ESM Fig 4. Type 2 diabetes prevalence in Indigenous youth, all ages, 1981-2015 19
ESM Fig 5. Type 2 diabetes prevalence trends in Indigenous youth, 7-19 years, 1986-2019 20
ESM REFERENCES 21

ESM METHODS

Search strategy – MEDLINE and Embase

#	Query
1	exp diabetes mellitus, type 2/
2	exp diabetes mellitus/
3	exp noncommunicable diseases/
4	type 2 diabetes.ab,ti.
5	type 2 diabetes mellitus.ab,ti.
6	type 2 dm.ab,ti.
7	t2dm.ab,ti.
8	"type ii diabet*".ab,ti.
9	"type-ii diabet*".ab,ti.
10	t2d.ab.
11	1 or 2 or 3 or 4 or 5 or 6 or 7 or 8 or 9 or 10
12	exp prevalence/
13	prevalence.ab,ti.
14	"prevalen*".ab,ti.
15	"screen*".ab,ti.
16	exp cross-sectional studies/
17	cross sectional.ab,ti.
18	cross-sectional.ab,ti.
19	12 or 13 or 14 or 15 or 16 or 17 or 18
20	11 and 19
21	exp Indigenous Peoples/
22	Indigenous Peoples.mp. [mp=ti, ab, hw, tn, ot, dm, mf, dv, kf, fx, dq, bt, nm, ox, px, rx, ui, sy, ux, mx]
23	indigenous population*.mp. [mp=ti, ab, hw, tn, ot, dm, mf, dv, kf, fx, dq, bt, nm, ox, px, rx, ui, sy, ux, mx]
24	indigenous.mp. [mp=ti, ab, hw, tn, ot, dm, mf, dv, kf, fx, dq, bt, nm, ox, px, rx, ui, sy, ux, mx]
25	indigen*.mp. [mp=ti, ab, hw, tn, ot, dm, mf, dv, kf, fx, dq, bt, nm, ox, px, rx, ui, sy, ux, mx]
26	aborig*.mp. [mp=ti, ab, hw, tn, ot, dm, mf, dv, kf, fx, dq, bt, nm, ox, px, rx, ui, sy, ux, mx]
27	aboriginal*.mp. [mp=ti, ab, hw, tn, ot, dm, mf, dv, kf, fx, dq, bt, nm, ox, px, rx, ui, sy, ux, mx]
28	aboriginal.mp. [mp=ti, ab, hw, tn, ot, dm, mf, dv, kf, fx, dq, bt, nm, ox, px, rx, ui, sy, ux, mx]
29	atsi.mp. [mp=ti, ab, hw, tn, ot, dm, mf, dv, kf, fx, dq, bt, nm, ox, px, rx, ui, sy, ux, mx]
30	australoid.mp. [mp=ti, ab, hw, tn, ot, dm, mf, dv, kf, fx, dq, bt, nm, ox, px, rx, ui, sy, ux, mx]
31	native people.mp. [mp=ti, ab, hw, tn, ot, dm, mf, dv, kf, fx, dq, bt, nm, ox, px, rx, ui, sy, ux, mx]
32	original inhabitants.mp. [mp=ti, ab, hw, tn, ot, dm, mf, dv, kf, fx, dq, bt, nm, ox, px, rx, ui, sy, ux, mx]
33	first people.mp. [mp=ti, ab, hw, tn, ot, dm, mf, dv, kf, fx, dq, bt, nm, ox, px, rx, ui, sy, ux, mx]
34	first nation.mp. [mp=ti, ab, hw, tn, ot, dm, mf, dv, kf, fx, dq, bt, nm, ox, px, rx, ui, sy, ux, mx]
35	torres strait*.mp. [mp=ti, ab, hw, tn, ot, dm, mf, dv, kf, fx, dq, bt, nm, ox, px, rx, ui, sy, ux, mx]
36	maori.mp. [mp=ti, ab, hw, tn, ot, dm, mf, dv, kf, fx, dq, bt, nm, ox, px, rx, ui, sy, ux, mx]
37	maori-polynesian.mp. [mp=ti, ab, hw, tn, ot, dm, mf, dv, kf, fx, dq, bt, nm, ox, px, rx, ui, sy, ux, mx]

38	melanesia*.mp. [mp=ti, ab, hw, tn, ot, dm, mf, dv, kf, fx, dq, bt, nm, ox, px, rx, ui, sy, ux, mx]
39	pacific island*.mp. [mp=ti, ab, hw, tn, ot, dm, mf, dv, kf, fx, dq, bt, nm, ox, px, rx, ui, sy, ux, mx]
40	inuit.mp. [mp=ti, ab, hw, tn, ot, dm, mf, dv, kf, fx, dq, bt, nm, ox, px, rx, ui, sy, ux, mx]
41	exp alaska natives/
42	alaska natives.mp. [mp=ti, ab, hw, tn, ot, dm, mf, dv, kf, fx, dq, bt, nm, ox, px, rx, ui, sy, ux, mx]
43	alaska* india*.mp. [mp=ti, ab, hw, tn, ot, dm, mf, dv, kf, fx, dq, bt, nm, ox, px, rx, ui, sy, ux, mx]
44	american indian.mp. [mp=ti, ab, hw, tn, ot, dm, mf, dv, kf, fx, dq, bt, nm, ox, px, rx, ui, sy, ux, mx]
45	north american indian.mp. [mp=ti, ab, hw, tn, ot, dm, mf, dv, kf, fx, dq, bt, nm, ox, px, rx, ui, sy, ux, mx]
46	indians, north american.mp. [mp=ti, ab, hw, tn, ot, dm, mf, dv, kf, fx, dq, bt, nm, ox, px, rx, ui, sy, ux, mx]
47	aotearoa.mp. [mp=ti, ab, hw, tn, ot, dm, mf, dv, kf, fx, dq, bt, nm, ox, px, rx, ui, sy, ux, mx]
48	tangata whenua.mp. [mp=ti, ab, hw, tn, ot, dm, mf, dv, kf, fx, dq, bt, nm, ox, px, rx, ui, sy, ux, mx]
49	metis.mp. [mp=ti, ab, hw, tn, ot, dm, mf, dv, kf, fx, dq, bt, nm, ox, px, rx, ui, sy, ux, mx]
50	native american.mp. [mp=ti, ab, hw, tn, ot, dm, mf, dv, kf, fx, dq, bt, nm, ox, px, rx, ui, sy, ux, mx]
51	native canadian.mp. [mp=ti, ab, hw, tn, ot, dm, mf, dv, kf, fx, dq, bt, nm, ox, px, rx, ui, sy, ux, mx]
52	21 or 22 or 23 or 24 or 25 or 26 or 27 or 28 or 29 or 30 or 31 or 32 or 33 or 34 or 35 or 36 or 37 or 38 or 39 or 40 or 41 or 42 or 43 or 44 or 45 or 46 or 47 or 48 or 49 or 50 or 51
53	animal.ab,ti.
54	animals.ab,ti.
55	rat.ab,ti.
56	rats.ab,ti.
57	mice.ab,ti.
58	mouse.ab,ti.
59	monkey.ab,ti.
60	monkeys.ab,ti.
61	trial.ab,ti.
62	trials.ab,ti.
63	case-control.ab,ti.
64	case control.ab,ti.
65	case series.ab,ti.
66	case report.ti.
67	genetic.ab,ti.
68	editorial.ab,ti.
69	covid-19.ab,ti.
70	coronavirus.ab,ti.
71	inpatient.ab,ti.
72	patient.ab,ti.
73	patients.ab,ti.
74	53 or 54 or 55 or 56 or 57 or 58 or 59 or 60 or 61 or 62 or 63 or 64 or 65 or 66 or 67 or 68 or 69 or 70 or 71 or 72 or 73

75	20 and 52
76	75 not 74
77	limit 76 to yr="2020-Current"
78	remove duplicates from 77

Search strategy – CINAHL

#	Query
S8	S7 NOT S6
S7	S1 AND S2 AND S3
S6	S4 OR S5
S5	AB Animal OR animals OR rat OR rats OR mice OR mouse OR monkey OR monkeys OR trial OR trials OR case-control OR case control OR case series OR genetic OR editorial OR covid-19 OR coronavirus OR inpatient OR patient OR patients
S4	TI Animal OR animals OR rat OR rats OR mice OR mouse OR monkey OR monkeys OR trial OR trials OR case-control OR case control OR case series OR case report OR genetic OR editorial OR covid-19 OR coronavirus OR inpatient OR patient OR patients
S3	Indigenous Peoples OR indigenous population* OR indigenous OR indigen* OR aborig* OR aboriginal* OR aboriginal OR atsi OR australoid OR native people OR original inhabitants OR first people OR first nation OR torres strait* OR maori OR maori-polynesian OR melanesia* OR pacific island* OR inuit OR alaska natives OR alaska* india* OR american indian OR north american indian OR indians, north American OR Aotearoa OR tangata whenua OR metis OR native american
S2	prevalence OR TI (prevalence OR prevalen* OR screen* OR cross sectional OR cross-sectional) OR AB (prevalence OR prevalen* OR screen* OR cross sectional OR cross-sectional) OR cross-sectional studies
S1	diabetes mellitus, type 2 OR diabetes mellitus OR noncommunicable diseases OR TI (type 2 diabetes OR type 2 diabetes mellitus OR type 2 dm OR t2dm) OR AB (type 2 diabetes OR type 2 diabetes mellitus OR type 2 dm OR t2dm OR t2d) OR TI type ii diabet* OR AB type ii diabet* OR TI type-ii diabet* OR AB type-ii diabet*

Diabetes in Indigenous Populations Special Interest Group of the International Diabetes Federation Diabetes Atlas Working Group Members

International team (listed alphabetically after Chair) of Indigenous and non-Indigenous professionals and researchers from the United States, Canada, Australia, Aotearoa New Zealand and Brazil.

1. Prof. Anthony Hanley (Chair), Department of Nutritional Sciences, University of Toronto, Toronto, ON, Canada and Department of Medicine and the Dalla Lana School of Public Health, University of Toronto, Toronto, ON, Canada and Leadership Sinai Centre for Diabetes, Mt. Sinai Hospital, Toronto, ON, Canada
2. Prof. Alex Brown, *Yuin Nation*, National Centre for Indigenous Genomics, Australian National University, Adelaide, SA, Australia and The Kids Research Institute Australia, Adelaide, SA, Australia
3. Dr Baiju Shah, Department of Medicine and Institute for Health Policy, Management and Evaluation, University of Toronto, Toronto, ON, Canada
4. Dr. Cheri Hotu, *Ngāti Maniapoto, Ngāti Ruanui,* Te Toka Tumai Auckland City Hospital, Te Whatu Ora, Aotearoa New Zealand
5. Dr. Courtney Claussen, *Cheyenne River Lakota*, School of Medicine and Health Sciences, University of North Dakota, Grand Forks, ND, USA and Johns Hopkins Bloomberg School of Public Health, Baltimore, MD, USA
6. Prof. Dianna Magliano, Baker Heart and Diabetes Institute, Melbourne, VIC, Australia and School of Public Health and Preventive Medicine, Monash University, Melbourne, VIC, Australia
7. Dr. Donald Warne, *Oglala Lakota*, Johns Hopkins Bloomberg School of Public Health, Baltimore, MD, USA
8. Dr. Elizabeth Barr, Menzies School of Health Research, Charles Darwin University, Darwin, NT, Australia and Baker Heart and Diabetes Institute, Melbourne, VIC, Australia
9. Dr. Emily Papadimos, Menzies School of Health Research, Charles Darwin University, Darwin, NT, Australia and Department of Endocrinology and Diabetes, Queensland Children's Hospital, Brisbane, QLD, Australia
10. Dr. Hiliary Monteith, Department of Nutritional Sciences, University of Toronto, Toronto, ON, Canada
11. The Late Dr Laercio Joel Franco, University of Sao Paulo, Brazil
12. Prof. Louise Maple Brown, Menzies School of Health Research, Charles Darwin University, Darwin, NT, Australia and Endocrinology Department, Royal Darwin Hospital, Darwin, NT, Australia
13. Dr. Odette Pearson, *Eastern Kuku-Yalanji and Zenadth Kes (Torres Strait Islander)*, South Australian Health and Medical Research Institute, University of Adelaide, Adelaide, SA, Australia and Faculty of Health and Medical Sciences, University of Adelaide, Adelaide, SA, Australia

ESM Fig 1. PRISMA study selection flowchart

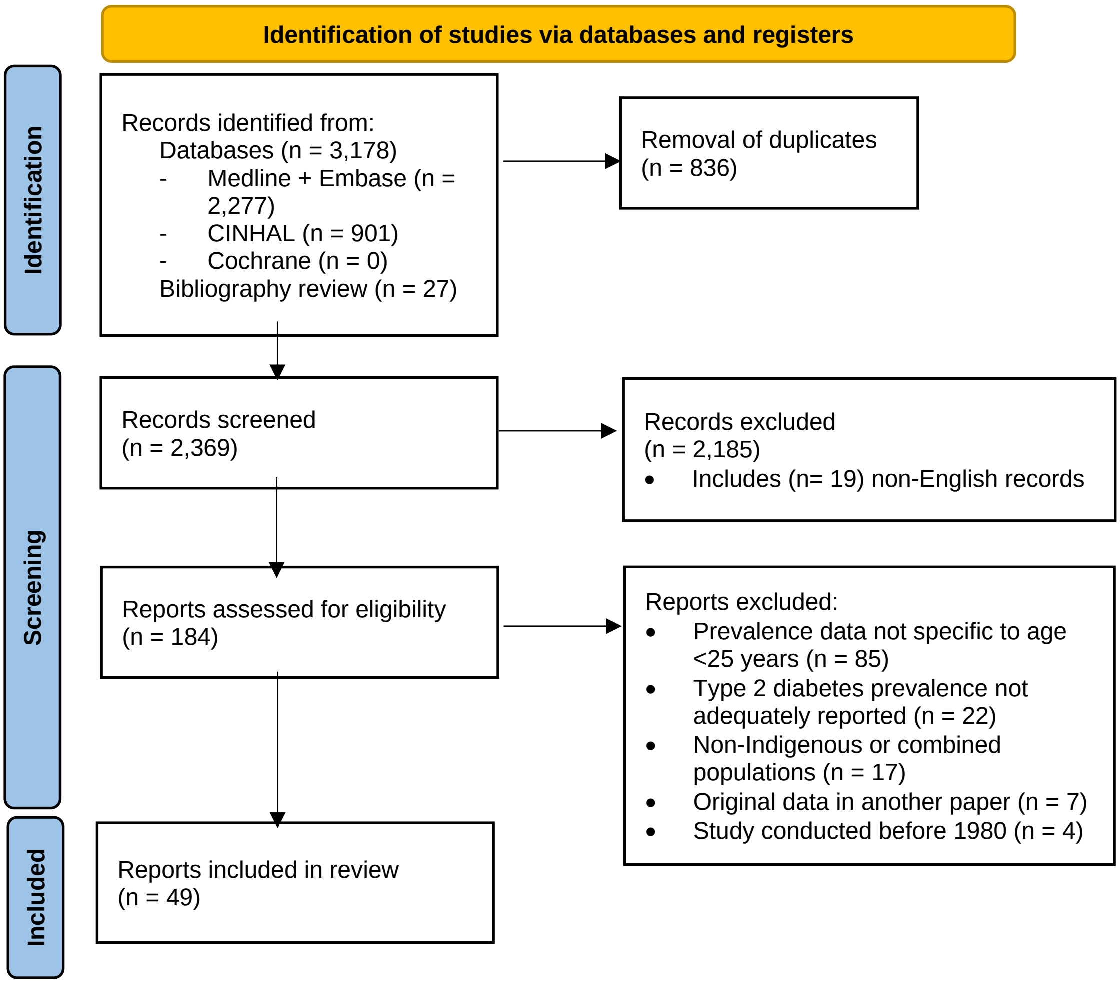

ESM Fig 2. Modified Newcastle-Ottawa Quality assessment scale

Quality score
The modified scale includes criteria that assesses the representativeness of Indigenous study populations, response rates, sample sizes, methods used to assess the outcome of interest, methods of assessing the outcome, and whether adjusted/standardized/crude prevalence is reported. The maximum score is 10 and final scores are defined as low quality (0-3 points), medium quality (4-7 points), or high quality (8-10 points).

Selection (Maximum 5 points):

1. Representative of the Indigenous population:
   a. Local Data: Reservation or Community-based. (3 points) eg: Indian Health Service Unit data, Community-based surveys, Tribal Census data, local electronic medical record data, local cohort data etc.
   b. Regional/Province/State Population Data (2 points) eg: Regional Indian Health Service data, State Telephone Surveys, State Census data, etc.
   c. National Level Data (1 point) eg: Registry, National level census data, etc.
   d. No description of the sample type (0 points)

2. Response rate
   a. The response rate is ≥80% (2 points)
   b. The response rate is ≥60% (1 point)
   c. The response rate is not applicable (1 point) eg: Electronic Medical Record data
   d. The response rate is ≤60%, or no description (0 points)

Outcome (Maximum 4 points):

1. Assessment of the outcome:
   a. Screening for diabetes prevalence (FPG, OGTT, HbA1c) (4 points)
   b. Only Capillary (fasting) (3 points)
   c. Administrative algorithm where at least two criteria are used (3 points)
   d. Record linkage or ICD code (2 points)
   e. Only Capillary (random) (1 point)
   f. Diabetes Medication (1 point)
   g. Self-report (1 point)
   h. No description (0 points)

Comparability (Maximum 1 point):

1. Is the reported prevalence adjusted/standardized?
   a. Yes (1 point)
   b. No, crude only (0 points)

Maximum score = 10 points

ESM TABLES

ESM Table 1. Data extraction

Author, Pub. Year	Year(s) of Study	Indigenous Nation/Tribe/ Population	Location/Region	Country	Age Group (years)	Prevalence (per 1000)	Male (per 1000)	Female (per 1000)	Sample Size (N)	Diagnosis	Crude(c) /standardised(s)
Pavkov, 2007[1]	1991-2003	Akimel O'odham & Tohono O'odham Indian	Gila River Community, Arizona	USA	5-14	33				OGTT	s[a]
	1978-1990				5-14	5					
	1991-2003				15-24	64					
	1978-1990				15-24	44					
Dabelea, 1998[2]	1987-1996	Akimel O'odham & Tohono O'odham Indian	Gila River Community, Arizona	USA	10-14		14	29	1103	OGTT, WHO 1985	c
					15-19		38	53	963		
Powell, 2019[3]	2009	Navajo	Arizona, New Mexico and Utah	USA	10-14	0.5			23104	administrative (ICD codes)	c
					15-19	2.2			26354		
					10-19	1.4	1.4	1.4	49458		
	2001				10-14	0.7			27107		
					15-19	2.4			23354		
					10-19	1.5	1.3	1.7	50461		
Kim, 1999[4]	1998	Navajo	Navajo Nation	USA	13-20	4			234	OGTT, WHO 1985	c
Dabelea, 2009[5]	2001	Navajo	NE Arizona, Utah, New Mexico	USA	0-9	0.1			47553	administrative (ICD codes)	c
					10-14	0.8			27107		
					15-19	2.8	2.1	2.6	23354		
Lee, 2004[6]	1999-2003	Cherokee Indian	Northeastern Oklahoma	USA	5-9	4	8	0	989	Fasting glucose, ADA 1997	c
					10-19	13	13	14			c
					5-19		11	10			c
Moore, 2003[7]	1999-2001	Northern Plains Indians	Montana and Wyoming	USA	<5	0			22886	medical records	c
					5-9	0.6					c
					10-14	3.5					c
					15-19	0.8					c
					<20		1	1.5	22886		c
Lawrence, 2021[8]	2017	American Indian	Multiple Regions[b]	USA	10-19	1.6			50558	administrative (ICD codes)	c
	2009					1.1			62573		c
	2001					1.2			64327		c
Nsiah-Kumi, 2013[9]	2007-2010	American Indian & Alaska Native	Great Plains Region	USA	5-18	10			201	OGTT, ADA 2010	c
Acton, 2002[10]	1990	American Indian & Alaska Native	Indian Health Reserves[c]	USA	<15	1.2	1.1	1.3		administrative (ICD codes)	c
					15-19	3.2	2.3	4.3			
					20-24	7.8	5.6	10.2			
	1991				<15	1.2	1.1	1.3			

					15-19	3.5	2.4	4.6			
					20-24	7.9	5.7	10.1			
	1992				<15	1.4	1.3	1.5			
					15-19	3.6	2.7	4.6			
					20-24	7.7	5.4	10.0			
	1993				<15	1.3	1.2	1.5			
					15-19	4.0	2.8	5.4			
					20-24	8.8	6.4	11.3			
	1994				<15	1.4	1.2	1.5			
					15-19	4.4	3.1	5.9			
					20-24	9.3	7.5	11.2			
	1995				<15	1.4	1.4	1.4			
					15-19	4.2	2.9	5.7			
					20-24	9.1	7.0	11.2			
	1996				<15	1.3	1.1	1.5			
					15-19	4.5	3.2	5.8			
					20-24	10.1	7.9	12.4			
	1997				<15	1.3	1.2	1.4			
					15-19	4.6	3.5	5.6			
					20-24	10.7	8.1	13.3			
	1998				<15	1.2	1.1	1.4			
					15-19	5.4	4.1	6.8			
					20-24	11.5	8.5	14.5			
Fagot-Campagna, 1999[11]	1997	American Indian & Alaska Native	Indian Health Reserves[d]	USA	15-19	4.6	3.4	5.7		administrative (ICD codes)	c
	1988					2.8	2.1	3.6			
Freeman, 1989[12]	1987	American Indian & Alaska Native	Indian Health Reserves[e]	USA	<24	1	1	1	28954	medical records	c
Valway, 1993[13]	1986-1987	American Indian & Alaska Native	Indian Health Reserves[f]	USA	0-14	1.3			1016815	medical record	c
Narayanan, 2010[14]	2006	Alaska Native: Aleut, Eskimo, Indian	Alaska	USA	0-14	0.7			41058	administrative (ICD codes)	c
					15-24	2.9			20489		
	1985				0-14	0.2			24961		
					15-24	0.3			17551		
Dai, 2024[15]	2012-2013	American Indian & Alaska Native	IHS regions: Alaska, East, Northern Plains, Pacific Coast, Southern Plains, Southwest	USA	<18	1.5	1.2	1.8		administrative (ICD codes)	c
					<10	0.2					
					10-19	4.2					
Dannenbaum, 2008[16]	2005	Cree	Eeyou Istchee (Eastern James Bay Cree), Quebec	Canada	10-19	3			1363	medical record, CDA 2003	c

Dannenbaum, 2005[17]	1999-2000	Cree	Eeyou Istchee (Eastern James Bay Cree), Quebec	Canada	10-19	0				FPG, HbA1c, CDA 1998	c
Delisle, 1993[18]	1989	Algonquin	Quebec	Canada	15-19	0	0	0	621	OGTT, WHO 1985	c
Zorzi, 2009[19]	2009	Tsimshian Nation	British Columbia[g]	Canada	6-18	0			224	OGTT, ADA 2007	c
Singer, 2014[20]	2014	Inuit	Naujaat (Repulse Bay), Nunavut	Canada	7-17	0			250	OGTT, CDA 1998	c
Dyck, 2012[21]	2003-2005	First Nations	Saskatchewan	Canada	<19		2.3	2.6		administrative algorithm	s[h]
	2000-2002						2.3	2.6			
	1997-1999						2.2	2.5			
	1994-1996						1.9	2.7			
	1991-1993						1.8	2.9			
	1988-1990						2.1	2.4			
	1985-1987						2	1.7			
	1982-1984						1.1	1.1			
	1980-1981						0.7	0.7			
	1980-2005						2	2.3			
Ralph-Campbell, 2009[22]	2006	Métis	Alberta	Canada	0-14	0	0	0	1662	Fasting glucose, CDA 2003	c
	2006				15-24	0	0	0	928		
	1998				0-14	1	0	1.2	1673		
	1998				15-24	0	0	0	656		
Oster, 2012[23]	1995	Status Aboriginal	Alberta	Canada	<20	1.3				administrative algorithm	c
	1996					1.3					
	1997					1.3					
	1998					1.4					
	1999					1.6					
	2000					1.6					
	2001					1.7					
	2002					2					
	2003					2.1					
	2004					2					
	2005					2.3					
	2006					2.5					
	2007					2.7			853733		
Shulman, 2020[24]	2014-2015	First Nations	Ontario	Canada	<5	0.7				administrative algorithm	c
	2014-2015				6-12	2.9					
	2014-2015				13-19	9.8					
	2014-2015				0-19	5.6	6.2	5.1			
	1995-1996				<19	1.7					
	1996-1997				<19	1.8					
	1997-1998				<19	1.8					

	1998-1999				<19	2.0					
	1999-2000				<19	2.3					
	2000-2001				<19	2.3					
	2001-2002				<19	2.5					
	2002-2003				<19	2.7					
	2003-2004				<19	3.1					
	2004-2005				<19	3.4					
	2005-2006				<19	3.7					
	2006-2007				<19	3.8					
	2007-2008				<19	3.7					
	2008-2009				<19	4.1					
	2009-2010				<19	4.6					
	2010-2011				<19	4.8					
	2011-2012				<19	4.8					
	2012-2013				<19	4.9					
	2013-2014				<19	5					
Smith, 2001[25]	2000	Beausoleil First Nation	Christian Island, Ontario	Canada	4-14	0			115	OGTT, CDA 1998	c
Evers, 1987[26]	1985	First Nations	Southwestern Ontario	Canada	0-4	0	0	0	1179	medical records	s[i]
					5-14	4	0	8			
					15-24	10	8	13			
Harris, 1997[27]	1993-1995	Anishininew (Ojibwa-Cree)	Sandy Lake, Ontario	Canada	10-19	44			728	OGTT, WHO 1985	c
Harris, 1996[28]	1978-1992	Anishininew (Ojibwa-Cree)	Sioux Lookout Zone, Northwestern Ontario	Canada	<16	2.5	0.7	4.2		administrative (ICD codes)	c
Fox, 1994[29]	1992	Anishininew (Ojibwa-Cree)	Sioux Lookout Zone, Northwestern Ontario	Canada	<15	1.7	1.1	2.2		administrative (ICD codes)	c
					15-24	7.2	3	14.4			c
Young, 1985[30]	1983	Anishininew (Ojibwa-Cree)	Sioux Lookout Zone, Northwestern Ontario	Canada	<15	0.5				administrative (ICD codes)	c
					15-24	4					c
Young, 2000[31]	1996-1997	Anishininew (Ojibwa-Cree)	St Theresa Point, Manitoba	Canada	4-19	11	0	23	719	Fasting glucose, ADA 1997	c
Sellers, 2024[32]	2009-2010	First Nations	Manitoba	Canada	0-17	2.9	1.8	3.9		Medical records	c
	2011-2012				0-17	3.6	2.3	5			
	2013-2014				0-17	4.4	2.6	6.2			
	2015-2016				0-17	4.7	3	6.4			
	2017-2018				0-17	5.2	3.7	6.8			
	2009-2010				7-17	4.8	3	6.7			
	2011-2012				7-17	6.3	4	8.7			
	2013-2014				7-17	7.8	4.7	11.1			
	2015-2016				7-17	8.3	5.4	11.3			

	2017-2018				7-17	9.1	6.4	11.8			
Bianchi, 2006[33]	2003	Toba Aboriginals	Toba, Mapic, Chelliyi and Fidelidad	Argentina	<25	0	0	0	385	Fasting or random glucose, ADA 1997	c
Joshy, 2009[34]	2007	Māori	Rotorua	Aotearoa New Zealand	0-9	1			45500	medical records	c
					10-19	2					c
Simmons, 1999[35]	1992-1995	Māori	South Auckland	Aotearoa New Zealand	<10	0			3910	self-report	c
					10-19	0.8			3614		
					<20	0.5			6277		
Simmons, 1994[36]	1992	Māori	Otara	Aotearoa New Zealand	<20	1			5606	self-report	c
Zimmet, 1992[37]	1982	Nauruan	Nauru	Nauru	8-19	6			625	OGTT, WHO 1985	c
King 1986[38]	1980	Cook Island Māori	Rarotonga	Cook Islands	20-24	6.9	0	10	2230	OGTT, WHO 1980	c
		Niuean	Niue	Niue	20-24	0	0	0		OGTT WHO 1980	c
Brimblecombe 2006[39]	2001-2002	Indigenous Australian	Northern Territory	Australia	15-24	0			332	Fasting glucose	c
Titmuss, 2022[40]	2016-2017	First Nations	Northern Australia	Australia	</=24	6.7	4.2	9.4	56882	administrative (ICD codes)	c
					<15	1.4			33335		c
					15-24	14.1			23547		c
			Top End		<15	0.8			12854		c
			Kimberley			0.8			4635		c
			Far North Queensland			1.8			11214		c
			Central Australia			2.8			4632		c
			Top End		15-24	13			7884		c
			Kimberley			20			2483		c
			Far North Queensland			8.3			9967		c
			Central Australia			31			3213		c
Valery, 2009[41]	2006	Torres Strait Islander	Torres Strait Islands	Australia	5-17	12			158	OGTT	c
O'Dea, 2008[42]	2003-2005	Both Aboriginal and TSI	Northern Territory	Australia	15-24	19	9.5	25	861	OGTT, WHO 1999	c
White, 1990[43]	1989	Australian Aboriginal	Great Sandy Desert, Fitzroy Valley	Australia	7-18	0			100	glucose, WHO 1985	c
Zhao, 2008[44]	2001-2005	Australian Aboriginal	Northern Territory	Australia	<15	2			29687	administrative (ICD codes)	c
McCulloch, 2003[45]	1998-2000	Australian Aboriginal	Far North Queensland	Australia	15-24	8	5	9	2862	self-report	c
		Torres Strait Islander			15-24	4	7	0			C
		Aboriginal and Islander			15-24	16	34	0			C

Cameron, 1986[46]	1982-1983	Australian Aboriginal	Bourke, New South Wales	Australia	20-24	41	26	58	294	OGTT, WHO 1980	C
Jeffries-Stokes, 2020[47]	2010-2012	Australian Aboriginal	Western Australia	Australia	<16	5			817	HbA1c	c
McDermott, 2000[48]	1987	Australian Aboriginal	Central Australia	Australia	15-24	NR	0	11	335	OGTT, WHO 1985	c
	1991				15-24	NR	42	12	331		c
	1995				15-24	NR	0	59	304		c
Hare, 2022[49]	2012-2019	Australian Aboriginal	Remote communities, Northern Territory	Australia	0-9	0.7	0.5	1	21267	administrative (ICD codes)	c
					10-19	14	7.9	20.5			c

USA: United States of America. OGTT: two-hour 75g oral glucose tolerance test. WHO: World Health Organisation. ICD: International Classification of Disease NE: Northeastern. ADA: American Diabetes Association. CDA: Canadian Diabetes Association. Blank cell indicates data not reported. [a]age- and sex-standardized using the 1985 Pima Indian population 5 years and older. [b]Four sites in Ohio; Colorado; five counties around Seattle, Washington; South Carolina; two sites in Hawaii and California; American Indian reservation-based populations in Arizona and New Mexico. [c]Alaska, Great Lakes, Northern Plains, Pacific, Southeast, Southern Plains, Southwest. [d]Arizona, Colorado, Nevada, New Mexico & Utah. [e]Washington, Oregon, and Idaho. [f]432 Indian Health Service facilities across the USA. [g]Gitga'at (Hartley Bay), Gitkxaahla (Kitkatla), Lax Kw'alaams (Port Simpson). [h]age-standardized using the 1991 estimated population of Canada. [i]age-standardizied using the 1985 estimated population of Canada.

ESM Table 2. Modified Newcastle-Ottawa Quality assessment results

**Author, publication year**	**Title**	**Representative (0-3)**	**Response Rate (0-2)**	**Outcome (0-4)**	**Comparability (0-1)**	**Total Score (0-10)**
**PASIFIKA**						
Zimmet, 1992	Hyperinsulinaemia in youth is a predictor of type 2 (non-insulin-dependent) diabetes mellitus.	3	1	4	0	8
King, 1986	Glucose tolerance in Polynesia. Population-based surveys in Rarotonga and Niue	3	2	4	1	10
**AOTEAROA NEW ZEALAND**						
Joshy G, 2008	Prevalence of diabetes in New Zealand general practice: the influence of ethnicity and social deprivation	2	1	3	1	7
Simons 1994	Prevalence of known diabetes in a multiethnic community.	2	2	1	1	6
Simmons, 1999	Prevalence of known diabetes in different ethnic groups in inner urban South Auckland.	2	2	1	1	6
**AUSTRALIA**						
Brimblecombe, 2006	Leanness and type 2 diabetes in a population of indigenous Australians	3	0	4	1	6
Titmuss, 2022	Youth-onset type 2 diabetes among First Nations young people in northern Australia: a retrospective, cross-sectional study	3	1	3	0	7
Valery, 2009	Prevalence of obesity and metabolic syndrome in Indigenous Australian youth	3	2	3	0	8
O'Dea 2008	Diabetes and cardiovascular risk factors in urban Indigenous adults: results from the DRUID study	3	0	4	0	7
White, 1990	Hyperinsulinaemia and impaired glucose tolerance in young Australian Aborigines	3	0	4	0	7
Zhao, 2008	Estimating chronic disease prevalence among the remote Aboriginal population of the Northern Territory using multiple data sources	2	1	3	0	6
McCulloch, 2003	Self-reported diabetes and health behaviors in remote indigenous communities in northern Queensland, Australia	3	0	1	0	4
Cameron, 1986	Diabetes mellitus in the Australian Aborigines of Bourke, New South Wales.	3	2	4	0	9
Jeffries-Stokes, 2020	Risk factors for renal disease and diabetes in remote Australia - findings from The Western Desert Kidney Health Project.	3	1	4	0	8
McDermott, 2000	Increase in prevalence of obesity and diabetes and decrease in plasma cholesterol in a central Australian aboriginal community.	3	1	4	0	8

Hare, 2022	Prevalence and incidence of diabetes among Aboriginal people in remote communities of the Northern Territory, Australia: a retrospective, longitudinal data-linkage study	3	1	3	0	7
**CANADA**						
Dannenbaum, 2008	Prevalence of diabetes and diabetes-related complications in FN communities in Northern Quebec (Eeyou Istchee), Canada	3	1	4	1	9
Dannenbaum, 2005	Undiagnosed diabetes in 2 Eeyou Istchee (Eastern James Bay Cree) communities: a population-based screening project	3	1	3	0	7
Smith, 2001	Type 2 diabetes in First Nation children: a collaborative effort to assess and prevent disease.	3	2	3	0	8
Zorzi, 2009	Prevalence of impaired glucose tolerance and the components of metabolic syndrome in Canadian Tsimshian Nation youth	3	2	4	0	9
Singer, 2014	Food consumption, obesity and abnormal glycaemic control in a Canadian Inuit community	3	0	4	0	7
Dyck, 2012	The epidemiology of diabetes mellitus among First Nations and non-First Nations children in Saskatchewan	2	1	3	1	7
Oster, 2012	Increasing rates of diabetes amongst status Aboriginal youth in Alberta, Canada	2	1	3	0	6
Sellers, 2024	Incidence and prevalence of type 2 diabetes in Manitoba children 2009–10 to 2017–18: First Nation versus all other Manitobans	2	1	3	0	6
Shulman, 2020	Prevalence, incidence and outcomes of diabetes in Ontario First Nations children: a longitudinal population-based cohort study	2	1	3	0	6
Evers, 1987	The prevalence of diabetes in Indians and Caucasians living in southwestern Ontario.	3	1	1	1	6
Ralph-Campbell, 2009	Increasing rates of diabetes and cardiovascular risk in Metis settlements in northern Alberta.	3	0	1	1	5
Young, 2000	Childhood obesity in a population at high risk for type 2 diabetes.	3	2	4	0	9
Delisle, 1993	Prevalence of non-insulin-dependent diabetes mellitus and impaired glucose tolerance in two Algonquin communities in Quebec	3	0	4	1	8
Young, 1985	Epidemiologic features of diabetes mellitus among Indians in Northwestern Ontario and Northeastern Manitoba	3	1	3	0	7
Harris, 1997	The prevalence of NIDDM and associated risk factors in Native Canadians	3	1	4	1	9
Harris, 1996	Non-insulin-dependent diabetes mellitus among First Nations children. New entity among First Nations people of Northwestern Ontario	3	1	0	1	5
Fox 1994	Diabetes among Native Canadians in Northwestern Ontario: 10 years later	3	1	3	1	8
**USA**						

Powell, 2019	Increasing burden of type 2 diabetes in Navajo youth: the SEARCH for diabetes in youth study.	3	1	2	0	6
Narayanan, 2010	Diabetes prevalence, incidence, complications and mortality among Alaska Native people 1985-2006	3	1	2	1	7
Lawrence, 2021	Trends in prevalence of type 1 and type 2 diabetes in children and adolescents in the US, 2001-2017	3	1	2	0	6
Pavkov, 2007	Changing patterns of type 2 diabetes incidence among Pima Indians	3	1	4	1	9
Moore, 2003	Three-year prevalence and incidence of diabetes among American India youth in Montana and Wyoming, 1999-2001	3	1	2	0	6
Dabelea, 2009	Diabetes in Navajo youth: prevalence, incidence, and clinical characteristics: the SEARCH for diabetes in youth study	3	1	2	0	6
Kim, 1999	Type 2 diabetes mellitus in Navajo adolescents	3	0	4	0	7
Lee, 2004	Type 2 diabetes and impaired fasting glucose in American Indians aged 5-40 years: the Cherokee diabetes study.	2	1	4	1	8
Fagot-Campagna, 1999	The public health epidemiology of type 2 diabetes in children and adolescents: a case study of American Indian adolescents in the Southwestern United States	2	1	2	0	5
Valway, 1993	Prevalence of diagnosed diabetes among American Indians and Alaska Natives, 1987. Estimates from a national outpatient data base	3	1	2	1	7
Acton, 2002	Trends in diabetes prevalence among American Indian and Alaska Native children, adolescents, and young adults	3	1	2	1	7
Freeman, 1989	Diabetes in American Indians of Washington, Oregon, and Idaho	3	1	2	1	7
Nsiah-Kumi, 2013	Diabetes, pre-diabetes and insulin resistance screening in Native American children and youth.	3	0	4	0	7
Dabelea, 1998	Increasing prevalence of type II diabetes in American Indian children	3	1	4	0	8
Dai, 2024	Prevalence of diagnosed type 1 and type 2 diabetes among American Indian and Alaska Native peoples in 2012–2013	3	1	3	0	7
**ARGENTINA**						
Bianchi, 2006	Epidemiology of renal and cardiovascular risk factors in Toba Aborigines	3	0	4	0	7

ESM FIGURES

ESM Fig 3. Diabetes prevalence according to diabetes subtype

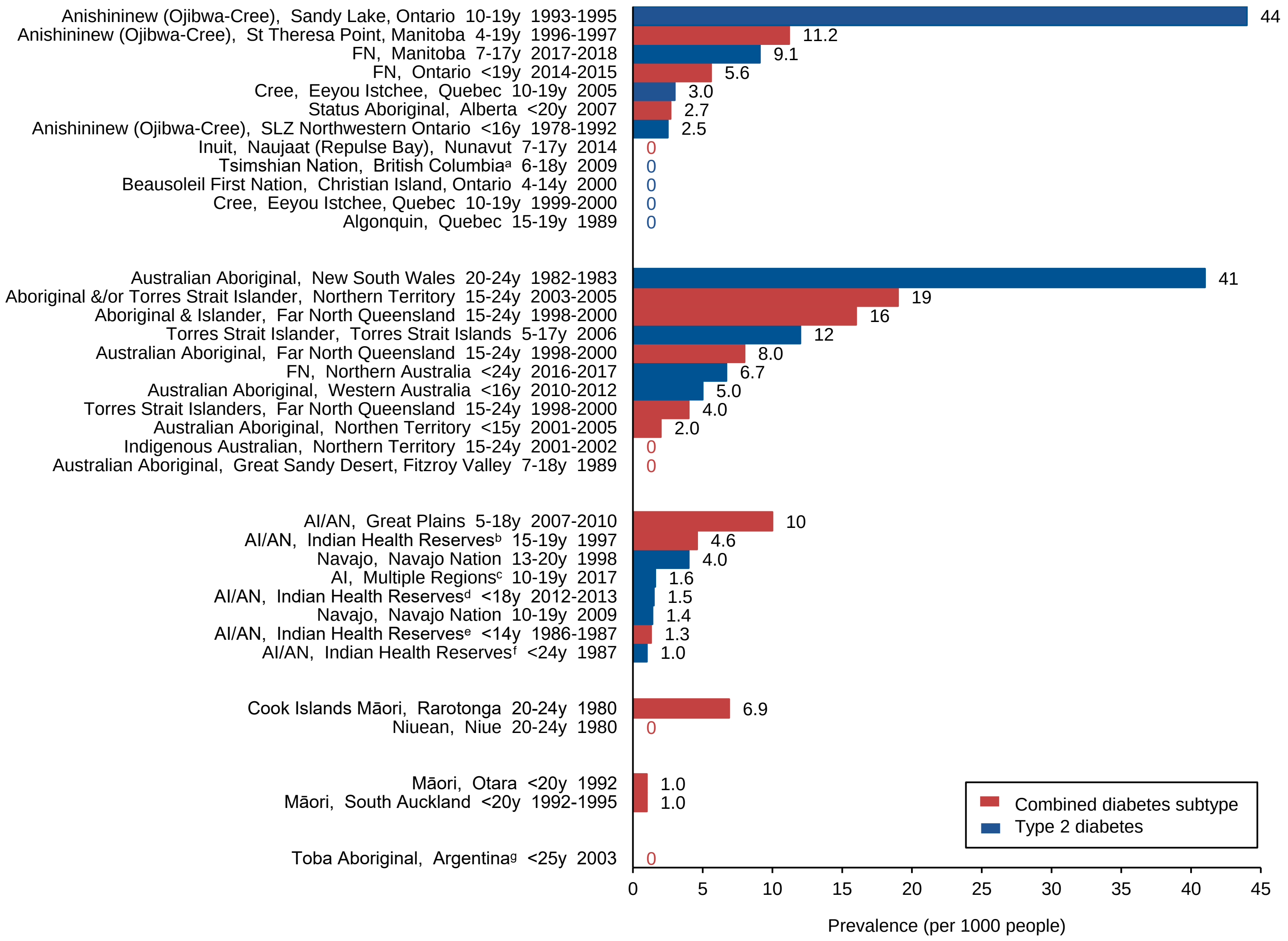

SLZ: Sioux Lookout Zone. AI: American Indian. AN: Alaskan Natives. [a]Gitga'at (Hartley Bay), Gitkxaahla (Kitkatla), Lax Kw'alaams (Port Simpson). [b]Arizona, Colorado, Nevada, New Mexico & Utah. [c]Four sites in Ohio; Colorado; five counties around Seattle, Washington; South Carolina; two sites in Hawaii and California; American Indian reservation-based populations in Arizona and New Mexico. [d]Six IHS regions: Alaska, East, Northern Plains, Pacific Coast, Southern Plains, Southwest. [e]432 Indian Health Service facilities across the USA. [f]Washington, Oregon, and Idaho. [g]Toba, Mapic, Chelliyi & Fidelidad.

ESM Fig 4. Type 2 diabetes prevalence in Indigenous youth, all ages, 1981-2015

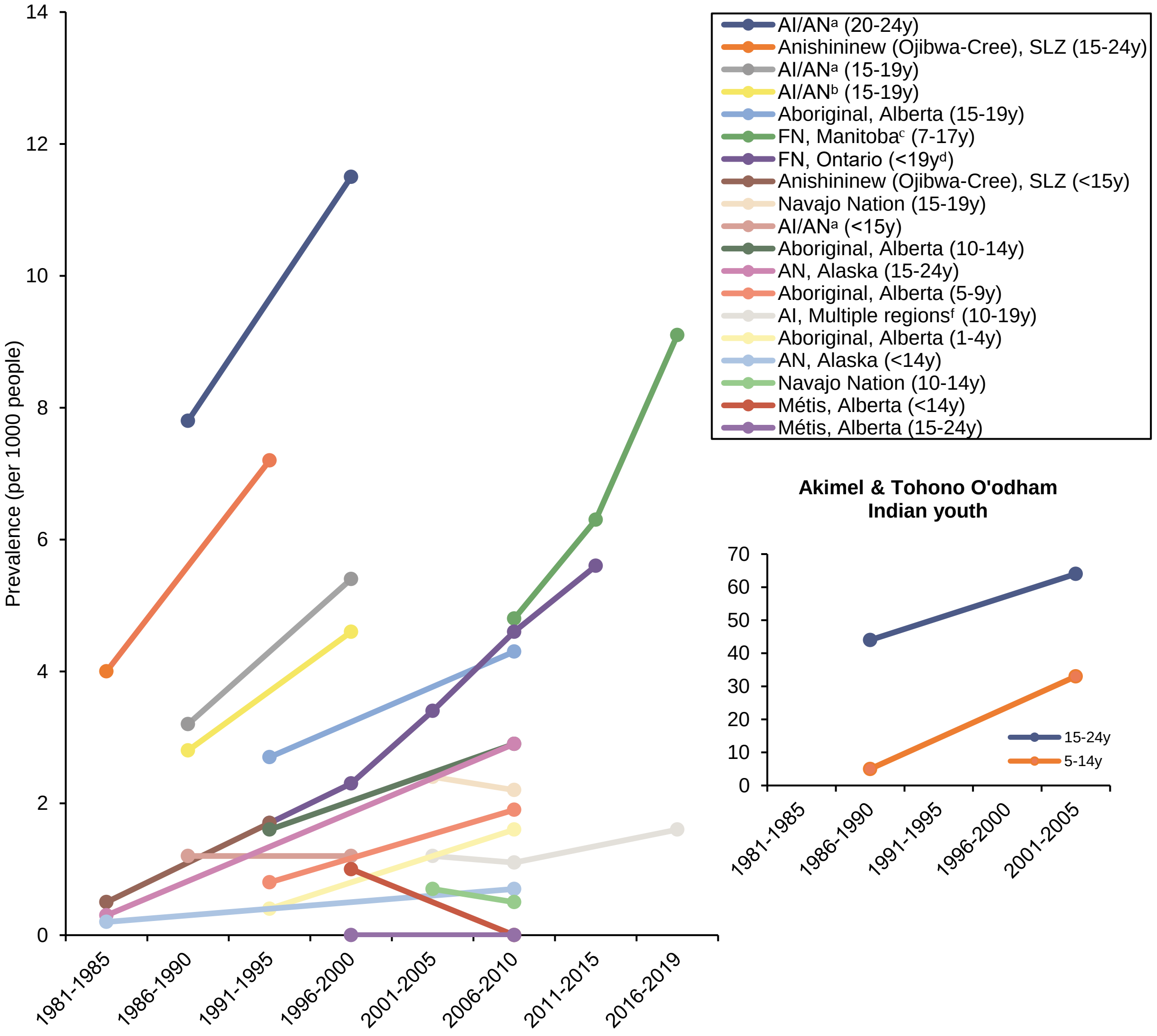

AI: American Indian. AN: Alaskan Natives. SLZ: Sioux Lookout Zone. [a]Alaska, Great Lakes, Northern Plains, Pacific, Southeast, Southern Plains, Southwest. [b]>78% of participants age 13 - 19 years. [c]Arizona, Colorado, Nevada, New Mexico & Utah. [d]Clinical registry data from Manitoba Diabetes Education Resource for Children and Adolescents (DER-CA). [e]Four sites in Ohio; Colorado; five counties around Seattle, Washington; South Carolina; two sites in Hawaii and California; American Indian reservation-based populations in Arizona and New Mexico.

ESM Fig 5. Type 2 diabetes prevalence trends in Indigenous youth, 7-19 years, 1986-2019

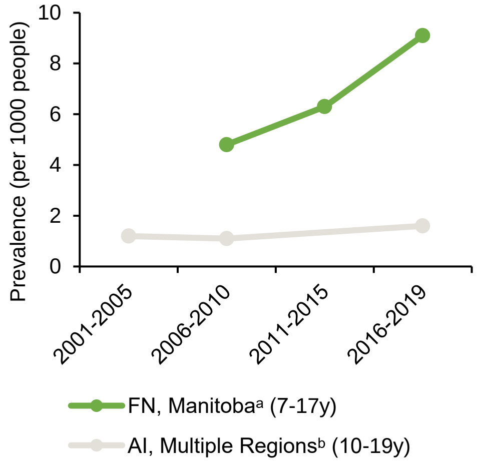

AI: American Indian. [a]Clinical registry data from Manitoba Diabetes Education Resource for Children and Adolescents (DER-CA). [b]Four sites in Ohio; Colorado; five counties around Seattle, Washington; South Carolina; two sites in Hawaii and California; American Indian reservation-based populations in Arizona and New Mexico.

ESM REFERENCES

[1] Pavkov ME, Hanson RL, Knowler WC, Bennett PH, Krakoff J, Nelson RG (2007) Changing patterns of type 2 diabetes incidence among Pima Indians. Diabetes Care 30(7): 1758-1763. 10.2337/dc06-2010

[2] Dabelea D, Hanson RL, Bennett PH, Roumain J, Knowler WC, Pettitt DJ (1998) Increasing prevalence of Type II diabetes in American Indian children. Diabetologia 41(8): 904-910. https://doi.org/10.1007/s001250051006

[3] Powell J, Isom S, Divers J, et al. (2019) Increasing burden of type 2 diabetes in Navajo youth: The SEARCH for diabetes in youth study. Pediatr Diabetes 20(7): 815-820. 10.1111/pedi.12885

[4] Kim C, McHugh C, Kwok Y, Smith A (1999) Type 2 diabetes mellitus in Navajo adolescents. The Western journal of medicine 170(4): 210-213

[5] Dabelea D, DeGroat J, Sorrelman C, et al. (2009) Diabetes in Navajo youth: prevalence, incidence, and clinical characteristics: the SEARCH for Diabetes in Youth Study. Diabetes care 32 Suppl 2: S141-S147. 10.2337/dc09-S206

[6] Lee ET, Begum M, Wang W, et al. (2004) Type 2 diabetes and impaired fasting glucose in American Indians aged 5-40 years: the Cherokee diabetes study. Ann Epidemiol 14(9): 696-704. 10.1016/j.annepidem.2003.10.013

[7] Moore KR, Harwell TS, McDowall JM, Helgerson SD, Gohdes D (2003) Three-year prevalence and incidence of diabetes among American Indian youth in Montana and Wyoming, 1999 to 2001. J Pediatr 143(3): 368-371. 10.1067/s0022-3476(03)00295-6

[8] Lawrence JM, Divers J, Isom S, et al. (2021) Trends in Prevalence of Type 1 and Type 2 Diabetes in Children and Adolescents in the US, 2001-2017. Jama 326(8): 717-727. 10.1001/jama.2021.11165

[9] Nsiah-Kumi PA, Lasley S, Whiting M, et al. (2013) Diabetes, pre-diabetes and insulin resistance screening in Native American children and youth. International journal of obesity 37(4): 540-545. 10.1038/ijo.2012.199

[10] Acton KJ, Burrows NR, Moore K, Querec L, Geiss LS, Engelgau MM (2002) Trends in diabetes prevalence among American Indian and Alaska native children, adolescents, and young adults. American journal of public health 92(9): 1485-1490

[11] Fagot-Campagna A, Burrows NR, Williamson DF (1999) The public health epidemiology of type 2 diabetes in children and adolescents: a case study of American Indian adolescents in the Southwestern United States. Clinica chimica acta; international journal of clinical chemistry 286(1-2): 81-95

[12] Freeman WL, Hosey GM, Diehr P, Gohdes D (1989) Diabetes in American Indians of Washington, Oregon, and Idaho. Diabetes care 12(4): 282-288

[13] Valway S, Freeman W, Kaufman S, Welty T, Helgerson SD, Gohdes D (1993) Prevalence of diagnosed diabetes among American Indians and Alaska Natives, 1987. Estimates from a national outpatient data base. Diabetes care 16(1): 271-276

[14] Narayanan ML, Schraer CD, Bulkow LR, et al. (2010) Diabetes prevalence, incidence, complications and mortality among Alaska Native people 1985-2006. Int J Circumpolar Health 69(3): 236-252. 10.3402/ijch.v69i3.17618

[15] Dai J, Niu X, Bullock A, Manson SM, O'Connell J, Jiang L (2024) Prevalence of Diagnosed Type 1 and Type 2 Diabetes Among American Indian and Alaska Native Peoples in 2012-2013. Diabetes Care 47(1): e1-e3. 10.2337/dc23-0930

[16] Dannenbaum D, Kuzmina E, Lejeune P, Torrie J, Gangbe M (2008) Prevalence of Diabetes and Diabetes-related Complications in First Nations Communities in Northern Quebec (Eeyou Istchee), Canada. Canadian Journal of Diabetes 32(1): 46-52. https://doi.org/10.1016/S1499-2671(08)21010-5

[17] Dannenbaum D, Torrie J, Noel F, Cheezo J, Sutherland L (2005) Undiagnosed diabetes in 2 Eeyou Istchee (Eastern James Bay Cree) communities: A population-based screening project. Canadian Journal of Diabetes 29: 397-402

[18] Delisle HF, Ekoé JM (1993) Prevalence of non-insulin-dependent diabetes mellitus and impaired glucose tolerance in two Algonquin communities in Quebec. CMAJ : Canadian Medical Association journal = journal de l'Association medicale canadienne 148(1): 41-47
[19] Zorzi A, Wahi G, Macnab AJ, Panagiotopoulos C (2009) Prevalence of impaired glucose tolerance and the components of metabolic syndrome in Canadian Tsimshian Nation youth. Canadian Journal of Rural Medicine 14(2): 61
[20] Singer J, Putulik Kidlapik C, Martin B, Dean HJ, Trepman E, Embil JM (2014) Food consumption, obesity and abnormal glycaemic control in a Canadian Inuit community. Clinical obesity 4(6): 316-323. 10.1111/cob.12074
[21] Dyck RF, Hayward MN, Harris SB (2012) Prevalence, determinants and co-morbidities of chronic kidney disease among First Nations adults with diabetes: results from the CIRCLE study. BMC nephrology 13: 57
[22] Ralph-Campbell K, Oster RT, Connor T, et al. (2009) Increasing rates of diabetes and cardiovascular risk in Métis Settlements in northern Alberta. International journal of circumpolar health 68(5): 433-442
[23] Oster RT, Johnson JA, Balko SU, Svenson LW, Toth EL (2012) Increasing rates of diabetes amongst status Aboriginal youth in Alberta, Canada. Int J Circumpolar Health 71(0): 1-7. 10.3402/ijch.v71i0.18501
[24] Shulman R, Slater M, Khan S, et al. (2020) Prevalence, incidence and outcomes of diabetes in Ontario First Nations children: a longitudinal population-based cohort study. CMAJ Open 8(1): E48-e55. 10.9778/cmajo.20190226
[25] Smith W, Gowanlock W, Babcock K (2001) Type 2 diabetes in First Nation children: A collaborative effort to assess and prevent disease. Paediatrics & child health 6(10): 755-759
[26] Evers S, McCracken E, Antone I, Deagle G (1987) The prevalence of diabetes in Indians and Caucasians living in southwestern Ontario. Canadian journal of public health = Revue canadienne de sante publique 78(4): 240-243
[27] Harris SB, Gittelsohn J, Hanley A, et al. (1997) The prevalence of NIDDM and associated risk factors in native Canadians. Diabetes care 20(2): 185-187
[28] Harris SB, Perkins BA, Whalen-Brough E (1996) Non-insulin-dependent diabetes mellitus among First Nations children. New entity among First Nations people of north western Ontario. Can Fam Physician 42: 869-876
[29] Fox C, Harris S, Whalen-Brough E (1994) Diabetes among Native Canadians in northwestern Ontario: 10 years later. Chronic Dis Can 15: 92-96
[30] Young TK, McIntyre LL, Dooley J, Rodriguez J (1985) Epidemiologic features of diabetes mellitus among Indians in northwestern Ontario and northeastern Manitoba. Canadian Medical Association journal 132(7): 793-797
[31] Young TK, Dean HJ, Flett B, Wood-Steiman P (2000) Childhood obesity in a population at high risk for type 2 diabetes. The Journal of pediatrics 136(3): 365-369
[32] Sellers EAC, McLeod L, Prior HJ, Dragan R, Wicklow BA, Ruth C (2024) Incidence and prevalence of type 2 diabetes in Manitoba children 2009-10 to 2017-18: First Nation versus all other Manitobans. Diabetes Res Clin Pract 208: 111097. 10.1016/j.diabres.2024.111097
[33] Bianchi ME, Farías EF, Bolaño J, Massari PU (2006) Epidemiology of renal and cardiovascular risk factors in Toba Aborigines. Renal failure 28(8): 665-670
[34] Joshy G, Porter T, Le Lievre C, Lane J, Williams M, Lawrenson R (2009) Prevalence of diabetes in New Zealand general practice: the influence of ethnicity and social deprivation. J Epidemiol Community Health 63(5): 386-390. 10.1136/jech.2008.078873
[35] Simmons D, Harry T, Gatland B (1999) Prevalence of known diabetes in different ethnic groups in inner urban South Auckland. The New Zealand medical journal 112(1094): 316-319

[36] Simmons D, Gatland B, Fleming C, Leakehe L, Scragg R (1994) Prevalence of known diabetes in a multiethnic community. The New Zealand medical journal 107(979): 219-222
[37] Zimmet PZ, Collins VR, Dowse GK, Knight LT (1992) Hyperinsulinaemia in youth is a predictor of type 2 (non-insulin-dependent) diabetes mellitus. Diabetologia 35(6): 534-541
[38] King H, Taylor R, Koteka G, et al. (1986) Glucose tolerance in Polynesia. Population-based surveys in Rarotonga and Niue. Med J Aust 145(10): 505-510. 10.5694/j.1326-5377.1986.tb139452.x
[39] Brimblecombe J, Mackerras D, Garnggulkpuy J, et al. (2006) Leanness and type 2 diabetes in a population of indigenous Australians. Diabetes Research and Clinical Practice 72(1): 93-99. 10.1016/j.diabres.2005.09.014
[40] Titmuss A, Davis EA, O'Donnell V, et al. (2022) Youth-onset type 2 diabetes among First Nations young people in northern Australia: a retrospective, cross-sectional study. The Lancet Diabetes & Endocrinology 10(1): 11-13. 10.1016/S2213-8587(21)00286-2
[41] Valery PC, Moloney A, Cotterill A, Harris M, Sinha AK, Green AC (2009) Prevalence of obesity and metabolic syndrome in Indigenous Australian youths. Obesity Reviews 10(3): 255-261. 10.1111/j.1467-789X.2008.00545.x
[42] O'Dea K, Cunningham J, Maple-Brown L, et al. (2008) Diabetes and cardiovascular risk factors in urban Indigenous adults: Results from the DRUID study. Diabetes Res Clin Pract 80(3): 483-489. 10.1016/j.diabres.2008.02.008
[43] White K, Gracey M, Schumacher L, Spargo R, Kretchmer N (1990) Hyperinsulinaemia and impaired glucose tolerance in young Australian aborigines. Lancet 335(8691): 735. 10.1016/0140-6736(90)90856-z
[44] Zhao Y, Connors C, Wright J, Guthridge S, Bailie R (2008) Estimating chronic disease prevalence among the remote Aboriginal population of the Northern Territory using multiple data sources. Aust N Z J Public Health 32(4): 307-313. 10.1111/j.1753-6405.2008.00245.x
[45] McCulloch B, McDermott R, Miller G, Leonard D, Elwell M, Muller R (2003) Self-reported diabetes and health behaviors in remote indigenous communities in northern queensland, australia. Diabetes care 26(2): 397-403
[46] Cameron WI, Moffitt PS, Williams DR (1986) Diabetes mellitus in the Australian aborigines of Bourke, New South Wales. Diabetes research and clinical practice 2(5): 307-314
[47] Jeffries-Stokes CA, Stokes AM, McDonald L, Evans S, Anderson Deceased L, Robinson PM (2020) Risk factors for renal disease and diabetes in remote Australia - findings from The Western Desert Kidney Health Project. Rural and remote health 20(2): 5440. 10.22605/RRH5440
[48] McDermott R, Rowley KG, Lee AJ, Knight S, O'Dea K (2000) Increase in prevalence of obesity and diabetes and decrease in plasma cholesterol in a central Australian aboriginal community. The Medical journal of Australia 172(10): 480-484
[49] Hare MJL, Zhao Y, Guthridge S, et al. (2022) Prevalence and incidence of diabetes among Aboriginal people in remote communities of the Northern Territory, Australia: a retrospective, longitudinal data-linkage study. BMJ Open 12(5): e059716. 10.1136/bmjopen-2021-059716
